# Supplementary material for: A deep proteome and transcriptome abundance atlas of 29 healthy human tissues
Source: Mol Syst Biol. 2019 Feb 18;15(2):e8503. doi: 10.15252/msb.20188503 (PMC6379049; doi:10.15252/msb.20188503)

Appendix to manuscript:

A deep proteome and transcriptome abundance atlas of 29 healthy human tissues

Dongxue Wang [1, #], Basak Eraslan [2, 2bis, #], Thomas Wieland [3], Björn Hallström [4], Thomas Hopf [3], Daniel Paul Zolg [1], Jana Zecha [1], Anna Asplund [5], Li-hua Li, Chen Meng[1], Martin Frejno [1], Tobias Schmidt, Karsten Schnatbaum[6], Mathias Wilhelm [1], Frederik Ponten [5], Mathias Uhlen [4], Julien Gagneur[2,] *, Hannes Hahne [3,*], Bernhard Kuster [1, 7, *]

1 Chair of Proteomics and Bioanalytics, Technische Universität München, Emil-Erlenmeyer-Forum 5, 85354 Freising, Germany

2 Computational Biology, Department of Informatics, Technical University of Munich, Boltzmannstr. 3, 85748, Garching bei München

2bis Quantitative Biosciences Munich, Gene Center, Department of Biochemistry, Ludwig Maximilian Universität, 81377 München, Germany

3 OmicScouts GmbH, Lise-Meitner-Str. 30, 85354 Freising, Germany

4 Science for Life Laboratory, KTH - Royal Institute of Technology, Stockholm, Sweden

5 Department of Immunology, Genetics and Pathology, Science for Life Laboratory, Uppsala University, Uppsala, Sweden

6 JPT Peptide Technologies GmbH, Berlin, Germany

7 Center For Integrated Protein Science Munich (CIPSM), Munich, Germany

# equal contribution

* Corresponding authors

To whom correspondence should be addressed

Email addresses

Bernhard Kuster, Chair of Proteomics and Bioanalytics, Technical University of Munich, Emil-Erlenmeyer-Forum 5, 85354 Freising, Germany, kuster@tum.de, phone: +49-8161-71-5696, fax: +49-8161-71-5931

Hannes Hahne, OmicScouts GmbH, Lise-Meitner-Str. 30, 85354 Freising, Germany, hannes.hahne@omicscouts.com, phone: +49-8161-976289-0, fax +49-8161-976289-1

Julien Gagneur, Computational Biology, Department of Informatics, Technical University of Munich, Boltzmannstr. 3, 85748, Garching bei München, Germany, gagneur@in.tum.de, phone: +49-89-289-19411

Content

1 Appendix Tables

Appendix Table S1. Digestion conditions for each protease

2 Appendix figures

Appendix Figure S1. Reproducibility of proteomic data

Appendix Figure S2. Reproducibility of transcriptomic data

Appendix Figure S3. Comparison of 29 tissue transcriptomic with transcriptomic data from the GTEx project

Appendix Figure S4. Comparison of shotgun proteomic data (this study) and targeted proteomic data (Edfors et al., Mol Sys Biol, 2016).

Appendix Figure S5. Comparison of protein quantitation of the tonsil proteome generated by different proteases and fragmentation methods.

Appendix Figure S6. Comparison of protein identifications of the ultra-deep tonsil proteome and the trypsin/HCD tonsil deep proteome

Appendix Figure S7. Myoglobin (MB): Example of a heart-specific tissue-enriched protein measured by shotgun proteomics, immunohistochemistry and targeted proteomics

Appendix Figure S8. Phosphoinositide-dependent kinase-1 (PDK1): Second example of a heart-specific tissue-enriched protein measured by shotgun proteomics, immunohistochemistry and targeted proteomics

Appendix Figure S9. Calcium-activated nucleotidase 1 (CANT1): Example of a prostate tissue-enhanced protein measured by shotgun proteomics, immunohistochemistry and targeted proteomics

Appendix Figure S10. Distribution of mRNA and protein copy numbers

Appendix Figure S11. Protein to mRNA abundance correlation plots of 29 tissues

Appendix Figure S12. Abundance distribution of all proteins detected in 29 tissues

Appendix Figure S13. Distribution of protein vs transcript correlations ('gene-wise' correlation across 29 tissues) of proteins expressed in at least 10 tissues

Appendix Figure S14. Distribution of protein vs transcript correlations ('gene-wise' correlation across 29 tissues) of proteins expressed in at least 20 tissues

Appendix Figure S15. Distribution of protein vs transcript correlations ('gene-wise' correlation across 29 tissues) of proteins expressed in all 29 tissues

Appendix Figure S16. Comparison of gene-wise protein and transcript correlations and protein abundance

Appendix Figure S17. Correlation analysis of transcripts and proteins of different levels of tissue-specificity classification

Appendix Figure S18. Influence of the sequence database on protein grouping

Appendix Figure S19. Isoform detection in the ultra-deep tonsil proteome and the deep trypsin/HCD tonsil proteome

Appendix Figure S20. Experimental vs synthetic peptide reference spectra of candidate SAAV peptides identified by Mascot

Appendix Figure S21. Experimental vs synthetic peptide reference spectra of candidate SAAV peptides identified by both Mascot and MaxQuant

Appendix Table S1. Digestion conditions for each protease

Proteases	**Resuspension buffer**	**Dilution buffer**	**Dilution volume**	**Protein: enzyme ratio**	**Temperature and time**
Trypsin (Roche)	50 mM HAc	50Mm Tris/HCl, pH 7.6	Add 4 volume to urea ~1.6M	50 : 1, add 2 times	37°C 4h, then overnight
LysC (Wako)	50mM Tris-HCl, pH 8.5	50 mM Tris/HCl, 1mM EDTA, pH 8.5	Add 8 volume to urea~0.8M	50 : 1, Add 2 times	37°C 4h, then overnight
ArgC (Promega)	50 mM Tris-HCl, 5mM $CaCl_2$, 2mM EDTA ,pH 7.6	50 mM Tris/HCl, 5mM $CaCl_2$,2mM EDTA(pH 7.6)<br><br>10× activation buffer: 50mM Tris/HCl, 50mM DTT,2mM EDTA, pH 7.6	Add 8 volume to urea ~ 0.8M, add activation buffer to a final concentration of 1×	60:1, Add 2 times	37°C 4h, then overnight
GluC (Promega)	Double-distilled water	50 mM Tris/HCl, pH 7.5	Add 8 volume to urea~0.8M	50 : 1, Add 2 times	37°C 4h, then overnight
AspN (Promega)	Double-distilled water	50Mm Tris/HCl, pH 7.5	Add 8 volume to urea~0.8M	100 : 1, Add 2 times	37°C 4h, then overnight
LysN (Promega)	50mM Tris-HCl,pH 8.0	50Mm Tris/HCl, pH 8.0	Add 8 volume urea~0.8M	100 : 1, Add 2 times	37°C 4h,then overnight
Chymotrypsin (Promega)	1 mM HCl	50 mM Tris/HCl, 10mM CaCl2, pH 8.0	Add 8 volume to urea~0.8M	50:1, Add 2 times	25°C 4h,then overnight

Appendix Figure S1. Reproducibility of proteomic data. Comparison of triplicate analysis of tonsil and liver tissues shows high correlation between replicates. A, heat map of the Spearman correlations of iBAQ values. B, scatter plots comparing replicate liver samples and protein identification in liver samples. C, scatter plots comparing replicate tonsil samples and protein identification in tonsils. Liver1 and Tonsil 1 were used in quantitative analyses of 29 tissues.

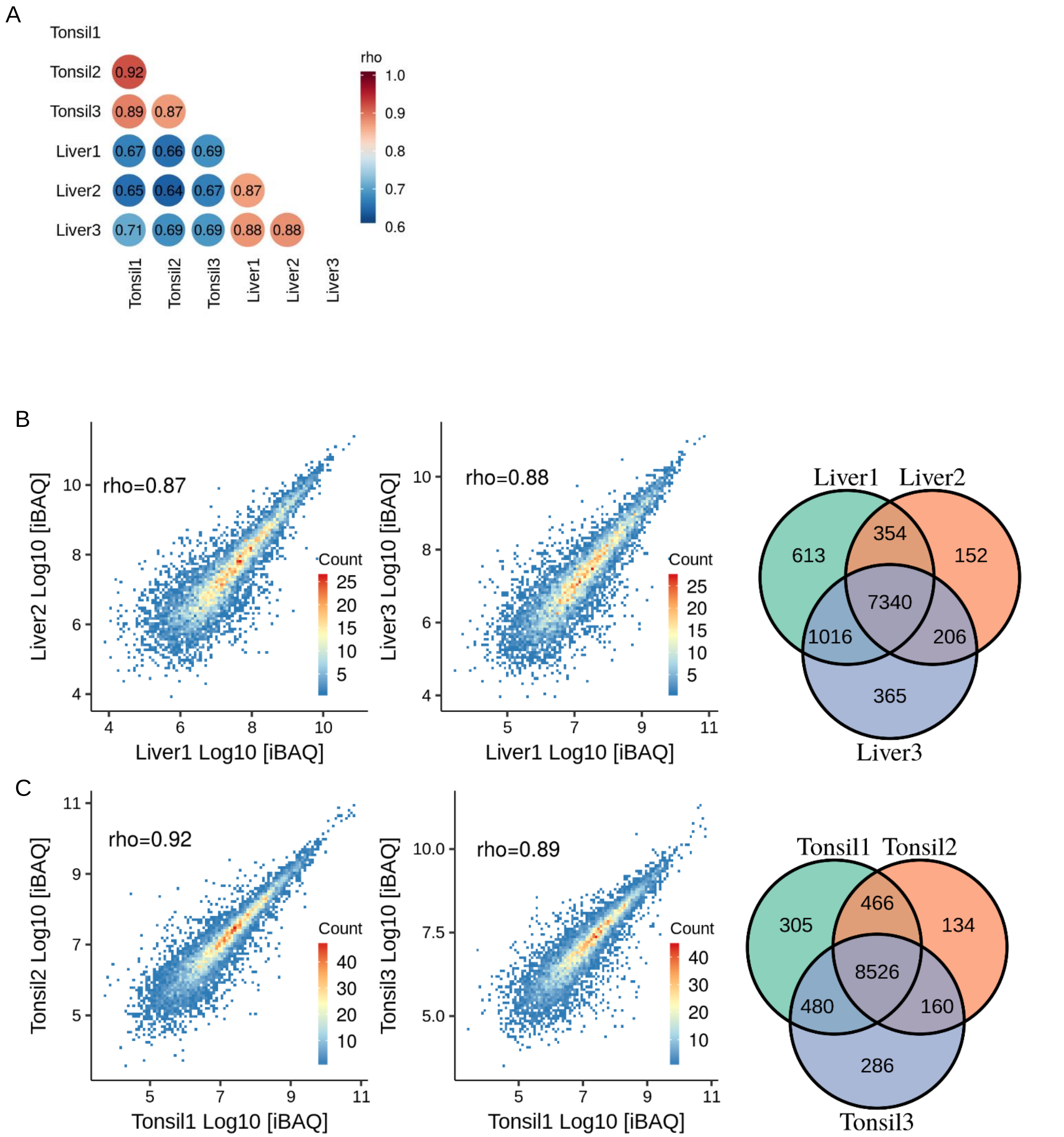

Appendix Figure S2. Reproducibility of transcriptomic data. Comparison of triplicate analysis of tonsil and liver tissues shows high correlation between replicates. A, heat map of the Spearman correlations of FPKM values.B, scatter plots comparing replicate liver samples and gene identification in liver samples. C, scatter plots comparing replicate tonsil samples and transcripts identification in tonsils. Please, note that only Liver 1 and Tonsil 1 have sample-matched proteomic and transcriptomic data which is why these were included in the expression atlas of 29 tissues.

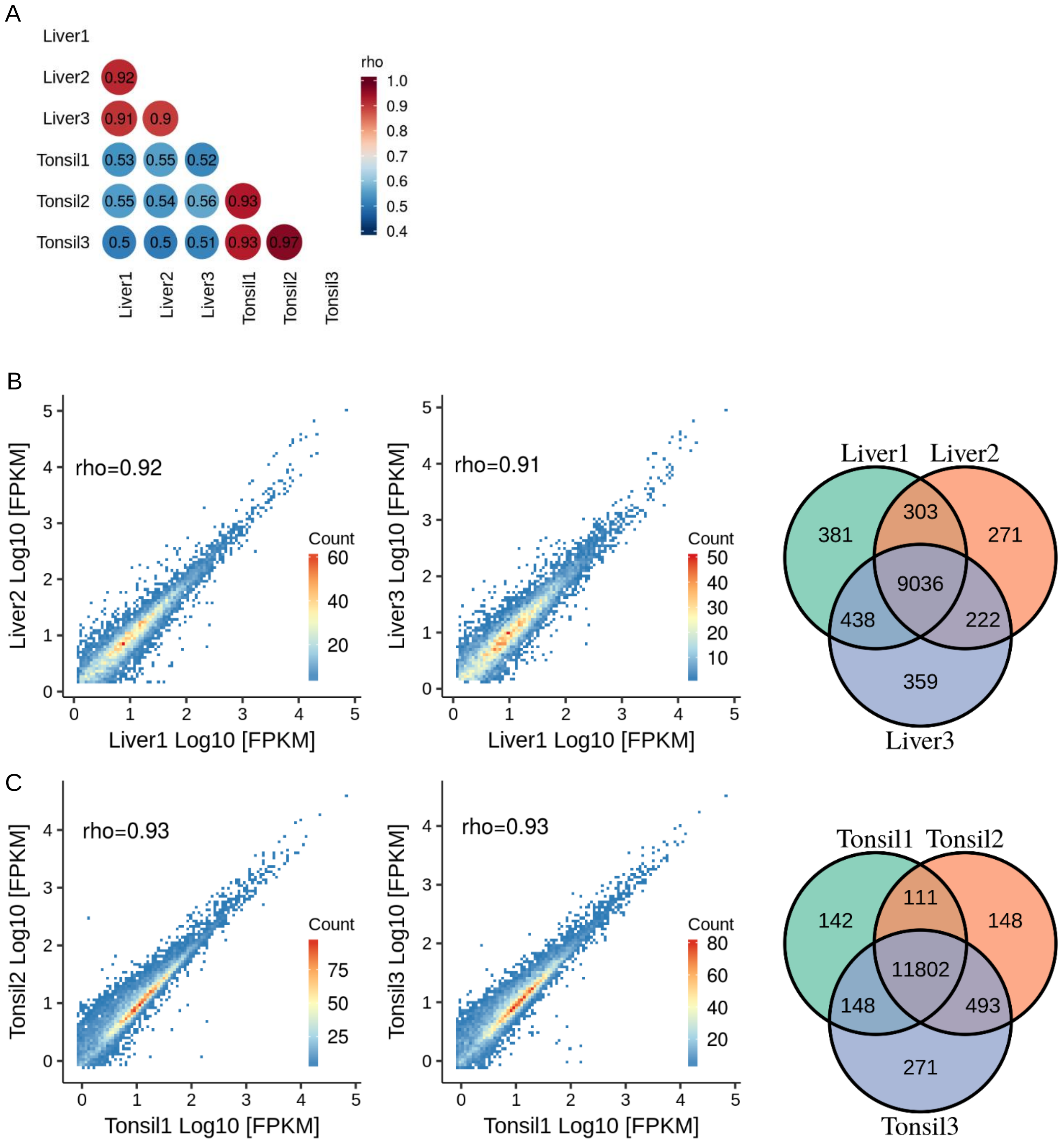

Appendix Figure S3. **Comparison of 29 tissue transcriptomes of the current study with transcriptomic data of the same (but not identical) tissues from the GTEx project.** Twelve tissues were analysed in both studies (adrenal gland, brain, fallopian tube, liver, lung, ovary, pancreas, prostate, spleen, stomach, testis, and thyroid; gtexportal.org, October 2018). Correlations are very high throughout indicating good overall quality of the transcriptomic data used in this study.

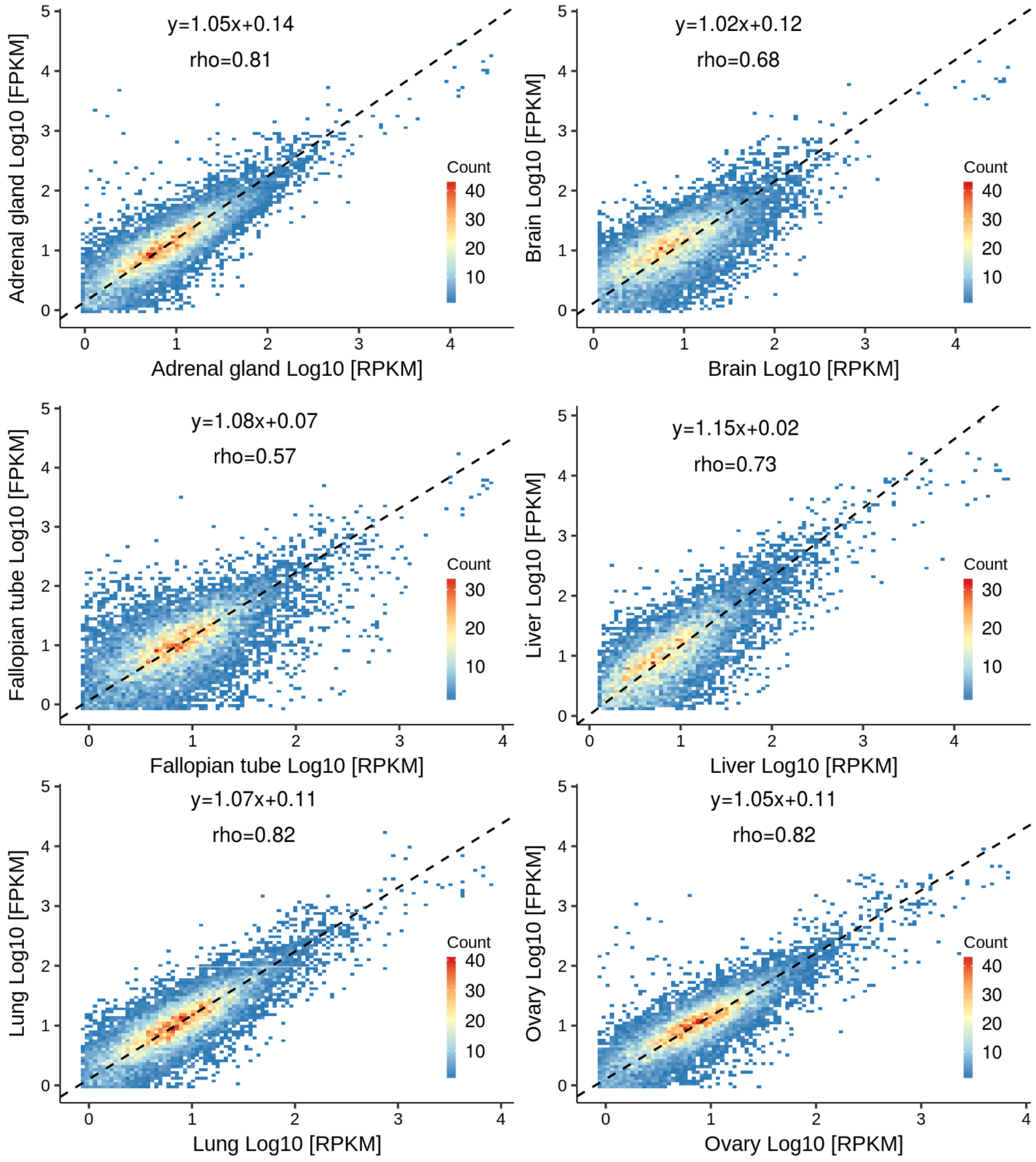

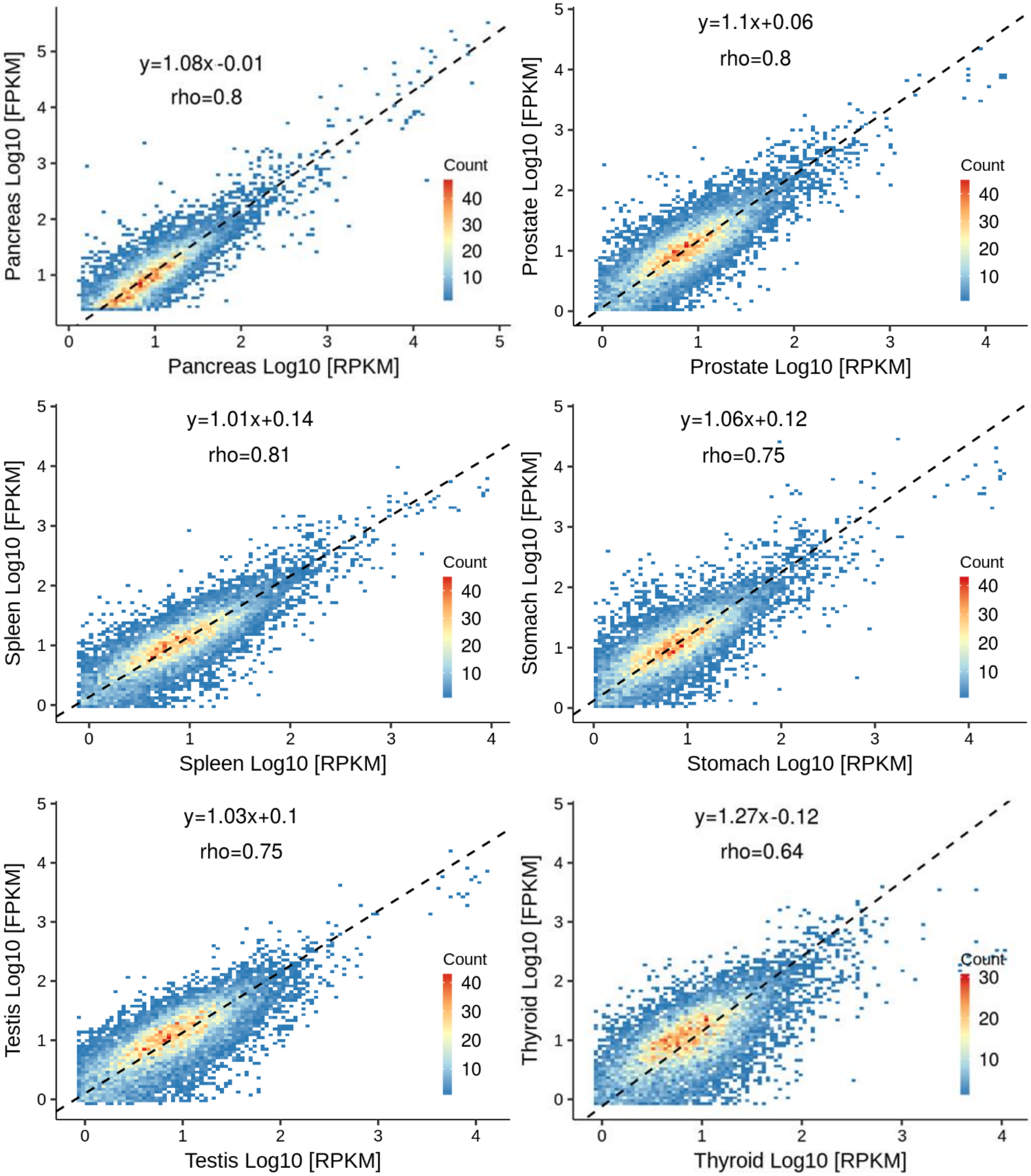
y=1.08x-0.01
rho=0.8
Pancreas Log10 [FPKM]
Pancreas Log10 [RPKM]
Count
y=1.1x+0.06
rho=0.8
Prostate Log10 [FPKM]
Prostate Log10 [RPKM]
Count
y=1.01x+0.14
rho=0.81
Spleen Log10 [FPKM]
Spleen Log10 [RPKM]
Count
y=1.06x+0.12
rho=0.75
Stomach Log10 [FPKM]
Stomach Log10 [RPKM]
Count
y=1.03x+0.1
rho=0.75
Testis Log10 [FPKM]
Testis Log10 [RPKM]
Count
y=1.27x-0.12
rho=0.64
Thyroid Log10 [FPKM]
Thyroid Log10 [RPKM]
Count

Appendix Figure S4. Comparison of shotgun proteomic data (this study) and targeted (PRM) proteomic data (Edfors et al., Mol Sys Biol, 2016). Edfors et al. analyzed ten different tissues (colon, duodenum, gall bladder, heart, kidney, liver, lung, prostate, spleen, tonsil) and 55 proteins. 52 of these proteins were also identified in the current study. Comparison of the copy numbers determined by PRM and our shotgun approach agreed very well. Depending on the tissue, the Spearman correlation coefficients were, on average, at 0.79 and, importantly, the slopes of the regression line were, on average, at 1.05 indicating that the shotgun data did not systematically over- or underestimate copy numbers (see appendix for correlation plots for all 10 tissues).

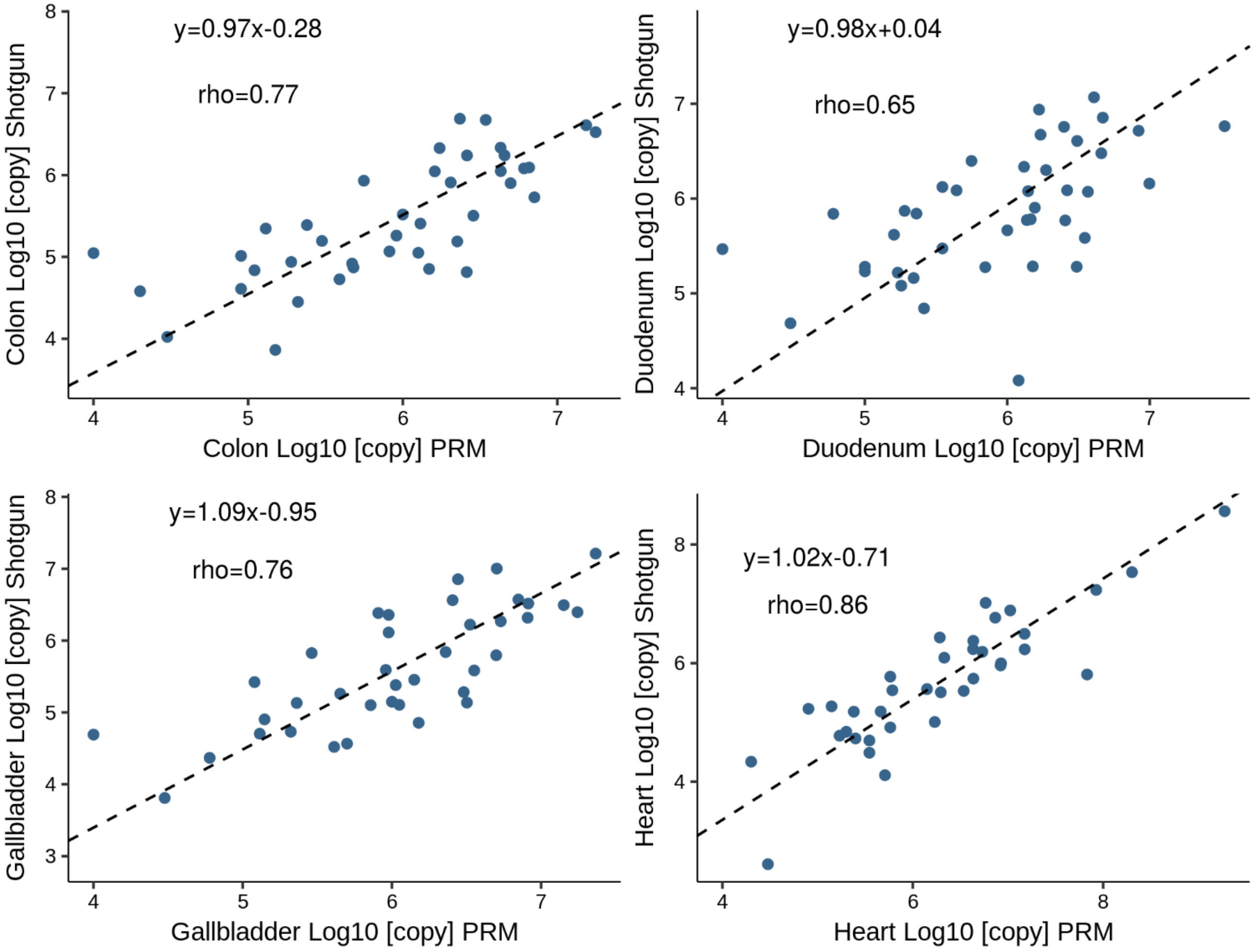

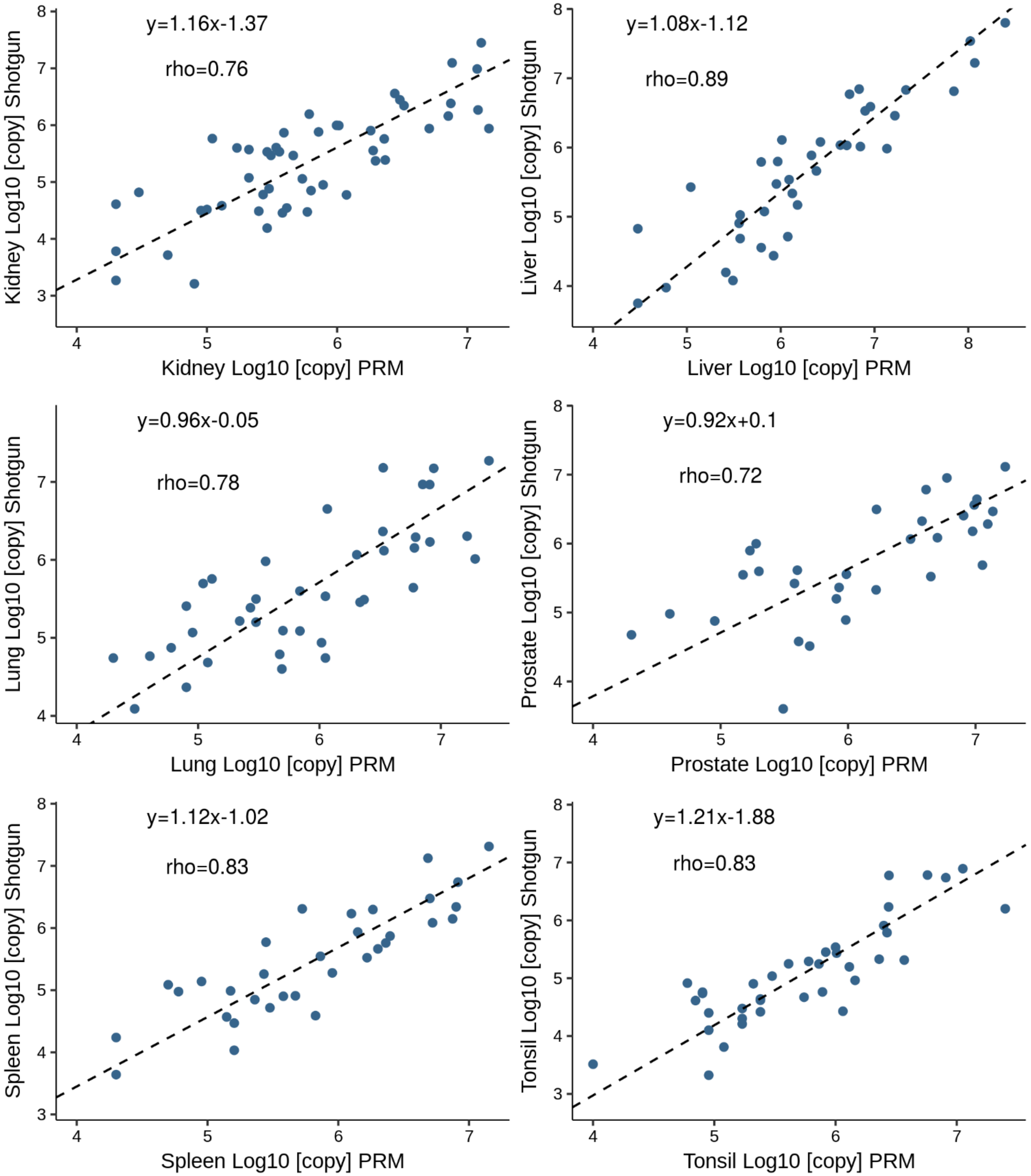

y=1.16x-1.37
rho=0.76
Kidney Log10 [copy] Shotgun
Kidney Log10 [copy] PRM
y=1.08x-1.12
rho=0.89
Liver Log10 [copy] Shotgun
Liver Log10 [copy] PRM
y=0.96x-0.05
rho=0.78
Lung Log10 [copy] Shotgun
Lung Log10 [copy] PRM
y=0.92x+0.1
rho=0.72
Prostate Log10 [copy] Shotgun
Prostate Log10 [copy] PRM
y=1.12x-1.02
rho=0.83
Spleen Log10 [copy] Shotgun
Spleen Log10 [copy] PRM
y=1.21x-1.88
rho=0.83
Tonsil Log10 [copy] Shotgun
Tonsil Log10 [copy] PRM

Appendix Figure S5. **Comparison of protein quantitation of the tonsil proteome generated by different proteases and fragmentation methods.** Protein intensity values were normalized by protein length. A, overview of length-normalized intensity correlations (Spearman's rank correlation). B, scatter plot comparing the total intensity of the ultra-deep proteome (across all proteases and fragmentation modes) and the intensity of the trypsin/HCD deep proteome data. C, Comparison of the ultra-deep proteome with the matching tonsil transcriptome. D, Comparison of the deep proteome (trypsin/HCD) with the matching tonsil transcriptome.

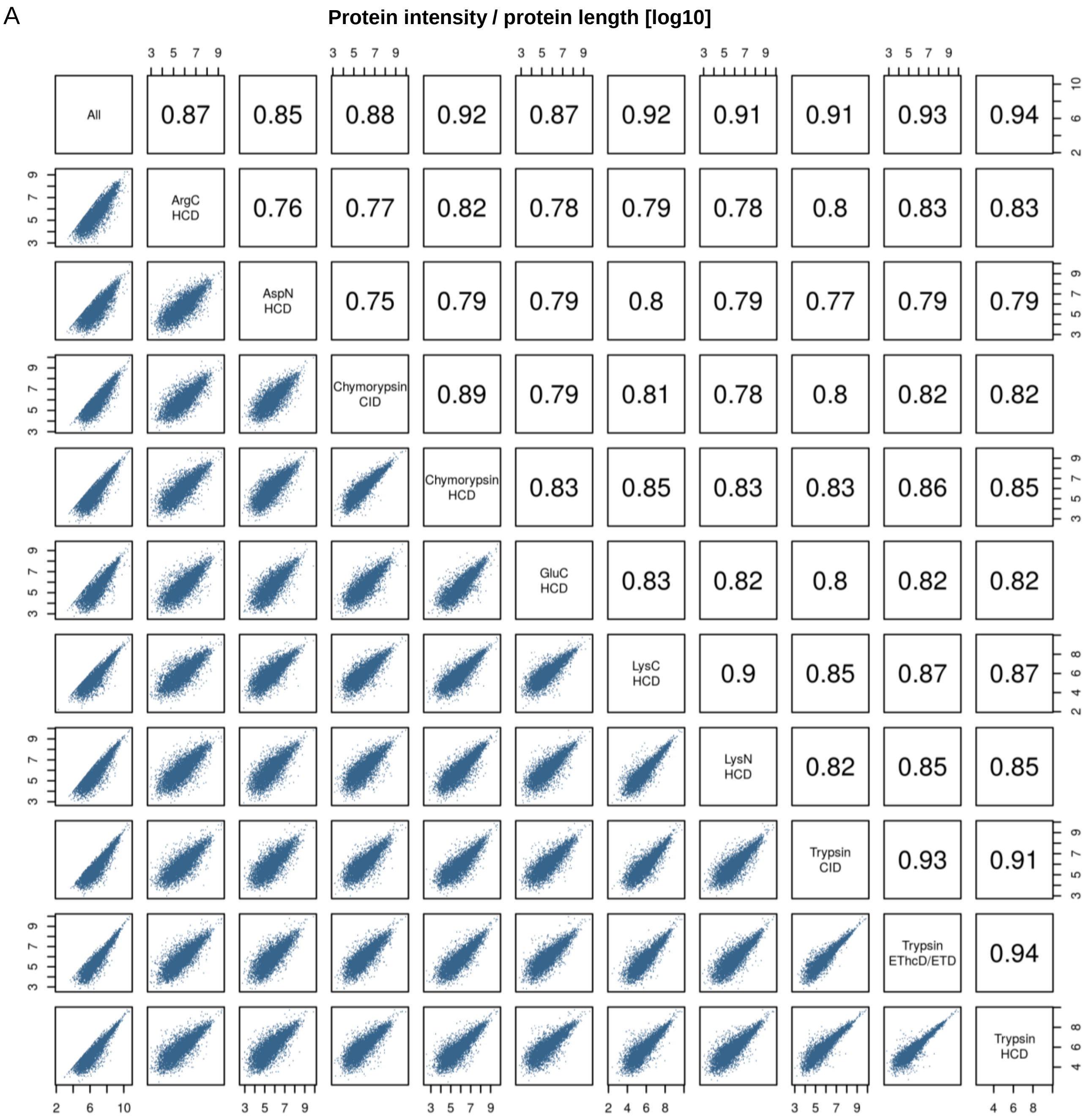

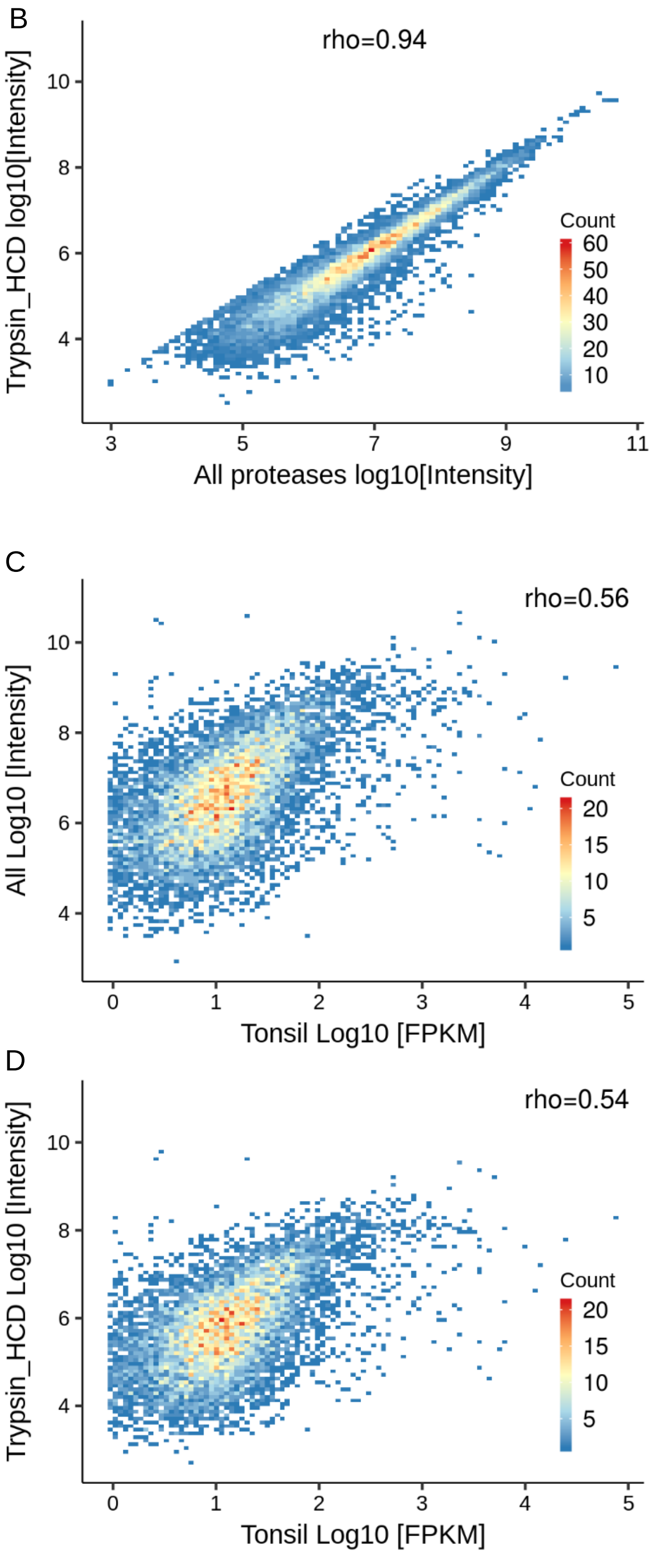
B
rho=0.94
Trypsin_HCD log10[Intensity]
All proteases log10[Intensity]
Count
60
50
40
30
20
10
C
rho=0.56
All Log10 [Intensity]
Tonsil Log10 [FPKM]
Count
20
15
10
5
D
rho=0.54
Trypsin_HCD Log10 [Intensity]
Tonsil Log10 [FPKM]
Count
20
15
10
5

Appendix Figure S6. **Comparison of protein identifications of the ultra-deep tonsil proteome and the trypsin/HCD tonsil deep proteome.** A, Comparison of protein identifications in the ulta-deep tonsil ('UltraTonsil') proteome, trypsin/HCD tonsil deep proteome ('Tonsil') and proteins not identified in the 'Tonsil' proteome, but in at least one other tissue ('OtherTissues'). B, comparison of the 504 proteins only identified in the ultra-deep tonsil proteome ('UltraTonsilOnly') and those identified with other proteases ('OtherProteases'). Of these 504 proteins, 282 were only identified because other proteases were used. But we cannot exclude the possibility that they could have been found in other tissues too if further proteases would have been included in the analysis of the other tissues. Therefore, these proteins are also not necessarily tissue-specific. The remaining 222 proteins from the in-depth tonsil could potentially represent tonsil-specific proteins.. C, transcript abundance distribution of all transcripts (grey, "Tonsil mRNA") and proteins detected in the ultra-deep tonsil proteome (blue, "All_proteases") and the trypsin/HCD tonsil proteome (orange, "Trypsin_HCD"), respectively. Please, note that the comparison was performed on gene symbol level. And, please, note that protein identifications in the ultra-deep tonsil proteome cannot benefit from the "match-between-runs" algorithm, while the approach was used for the trypsin/HCD dataset (tonsils + 28 other tissues). The data shows that while the ultra-deep tonsil proteome identified more proteins, it did not identify significantly more low abundance proteins than the standard trypsin-HCD workflow.

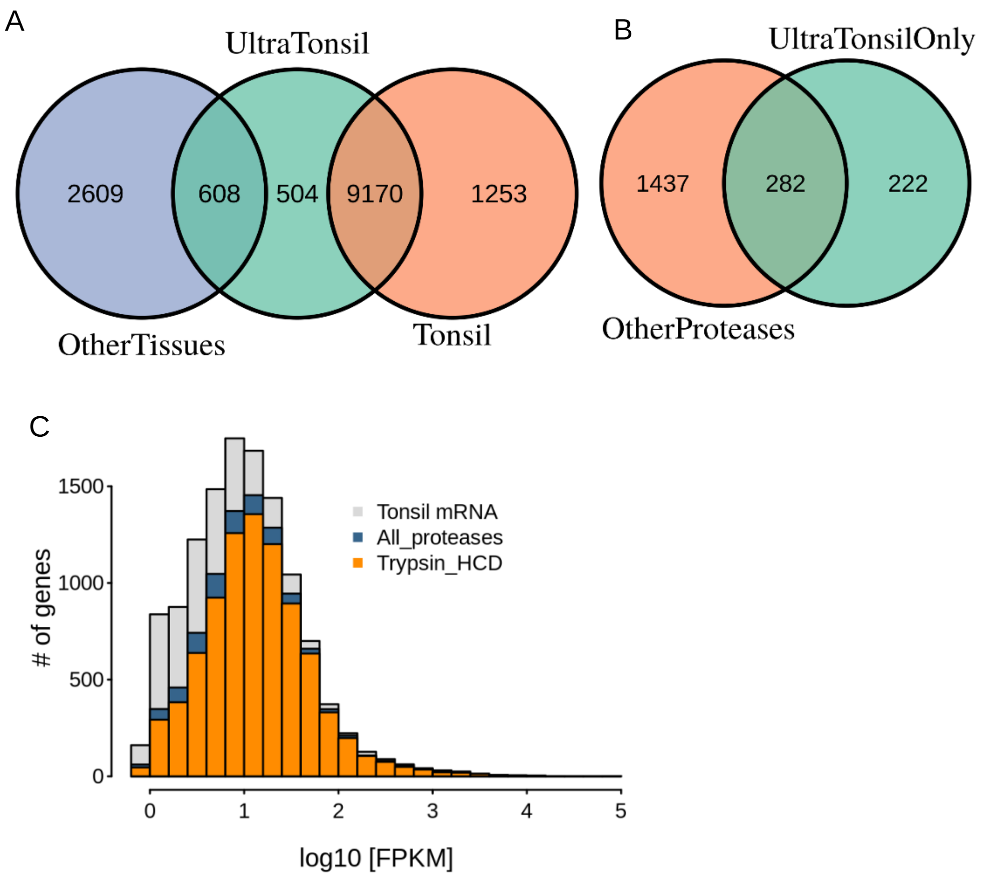

Appendix Figure S7. Myoglobin (MB): Second example of a heart-specific tissue-enriched protein measured by shotgun proteomics, immunohistochemistry and targeted proteomics. Also MB is a heart-specific protein and the plot shows the iBAQ values (upper panel), the immunohistochemistry (IH) score (middle panel, soure: Human Protein Atlas) as well as protein copies determined via PRM (bottom panel, source: Edfors et al., 2016). Please, note that an IH score of 0 means that the protein was not detected and a score of 100 represents a very intense, robust IH detection of the protein. The PRM analysis covered ten tissues.

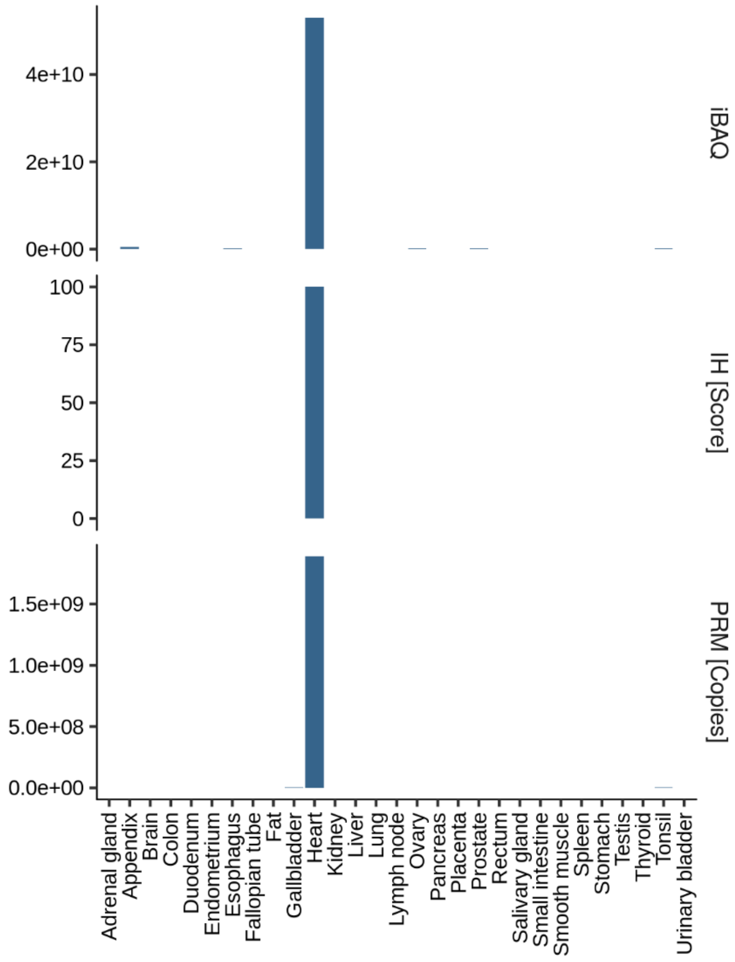

Appendix Figure S8. **Phosphoinositide-dependent kinase-1 (PDK1): Example of a heart-specific tissue-enriched protein measured by shotgun proteomics, immunohistochemistry and targeted proteomics**. PDK1 is a heart-specific protein and the plot shows the iBAQ values (upper panel), the immunohistochemistry (IH) score (middle panel, soure: Human Protein Atlas) as well as protein copies determined via PRM (bottom panel, source: Edfors et al., 2016). Please, note that an IH score of 0 means that the protein was not detected and a score of 100 represents a very intense, robust IH detection of the protein. The PRM analysis covered ten tissues.

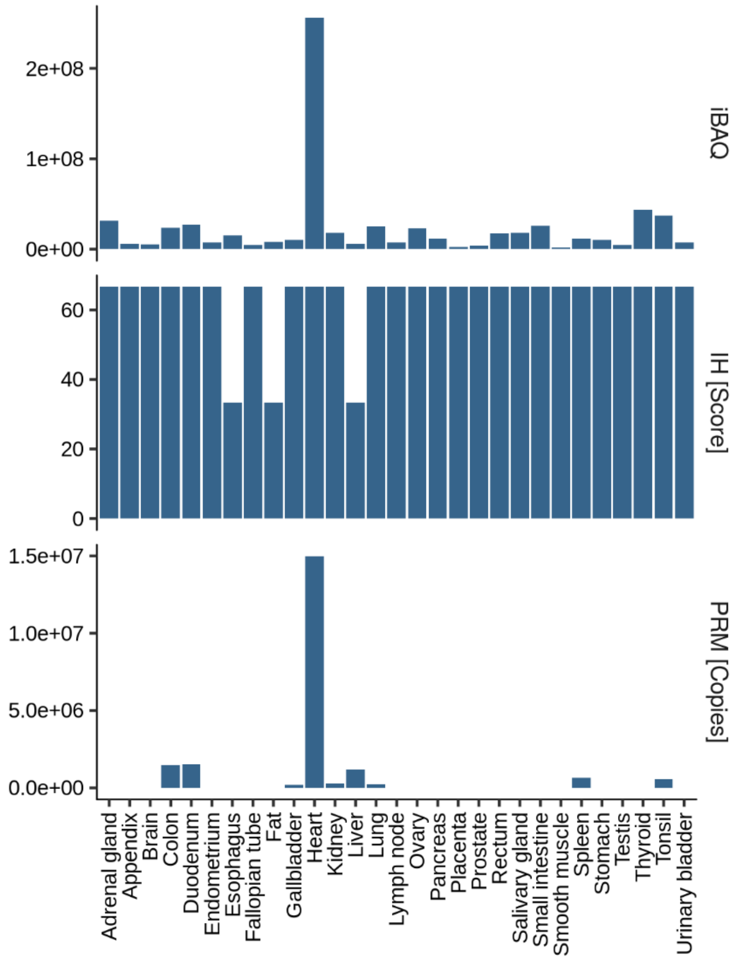

Appendix Figure S9. **Calcium-activated nucleotidase 1 (CANT1): Example of a prostate tissue-enhanced protein measured by shotgun proteomics, immunohistochemistry and targeted proteomics.** CANT1 is a protein highly expressed in prostate and the plot shows the iBAQ values (upper panel), the immunohistochemistry (IH) score (middle panel, soure: Human Protein Atlas) as well as protein copies determined via PRM (bottom panel, source: Edfors et al., 2016). Please, note that an IH score of 0 means that the protein was not detected and a score of 100 represents a very intense, robust IH detection of the protein. The PRM analysis covered ten tissues.

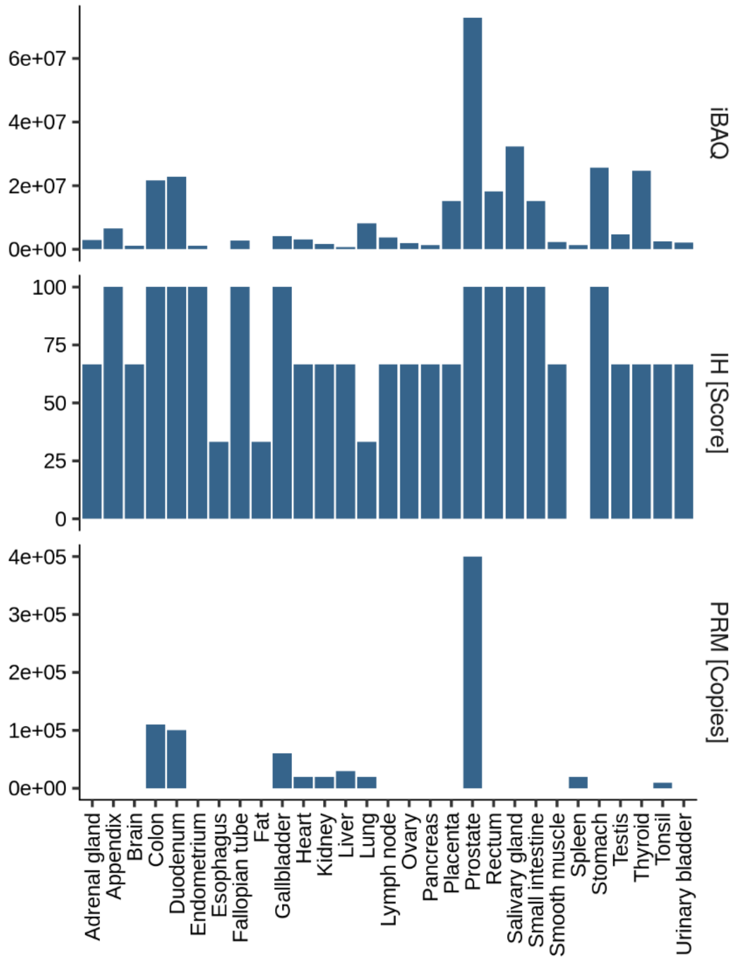

Appendix Figure S10. Distribution of mRNA and protein copy numbers. A, global distribution of transcript and protein copies in all tissues. It is apparent that the dynamic range of protein expression exceeds that of mRNA expression. B, scatter plot comparing two "protein concentration" measures, iBAQ and protein copy numbers, of the brain proteome as example. Protein copies were calculated based on the ProteomicRuler (Wiśniewski et al., 2014). C, scatter plot comparing FPKM and mRNA copies. Transcript copies were calculated according to the ProteomicRuler and the assumption that the cellular mRNA mass represents about 1-3% of the total cellular RNA mass (Melnikov et al., 2012).

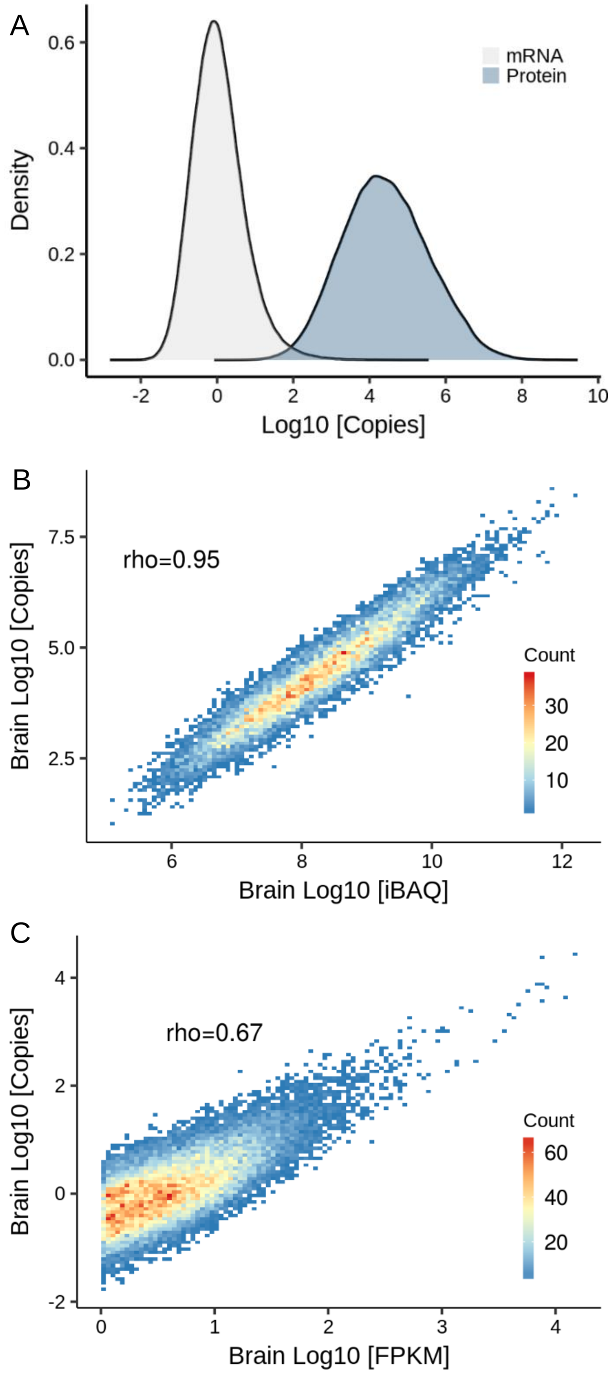

Appendix Figure S11. Protein to mRNA abundance correlation plots of all 29 tissues investigated in this study.

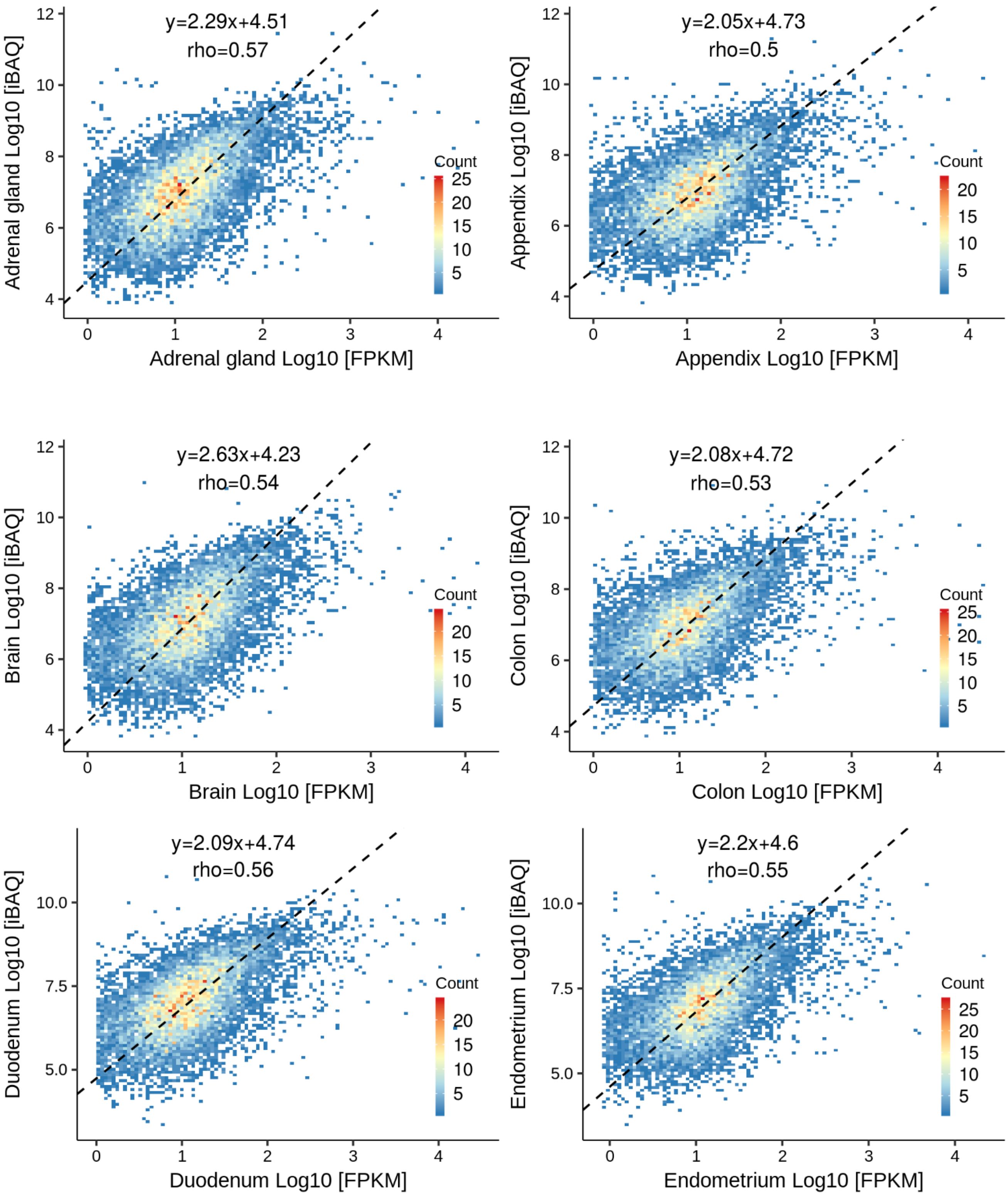

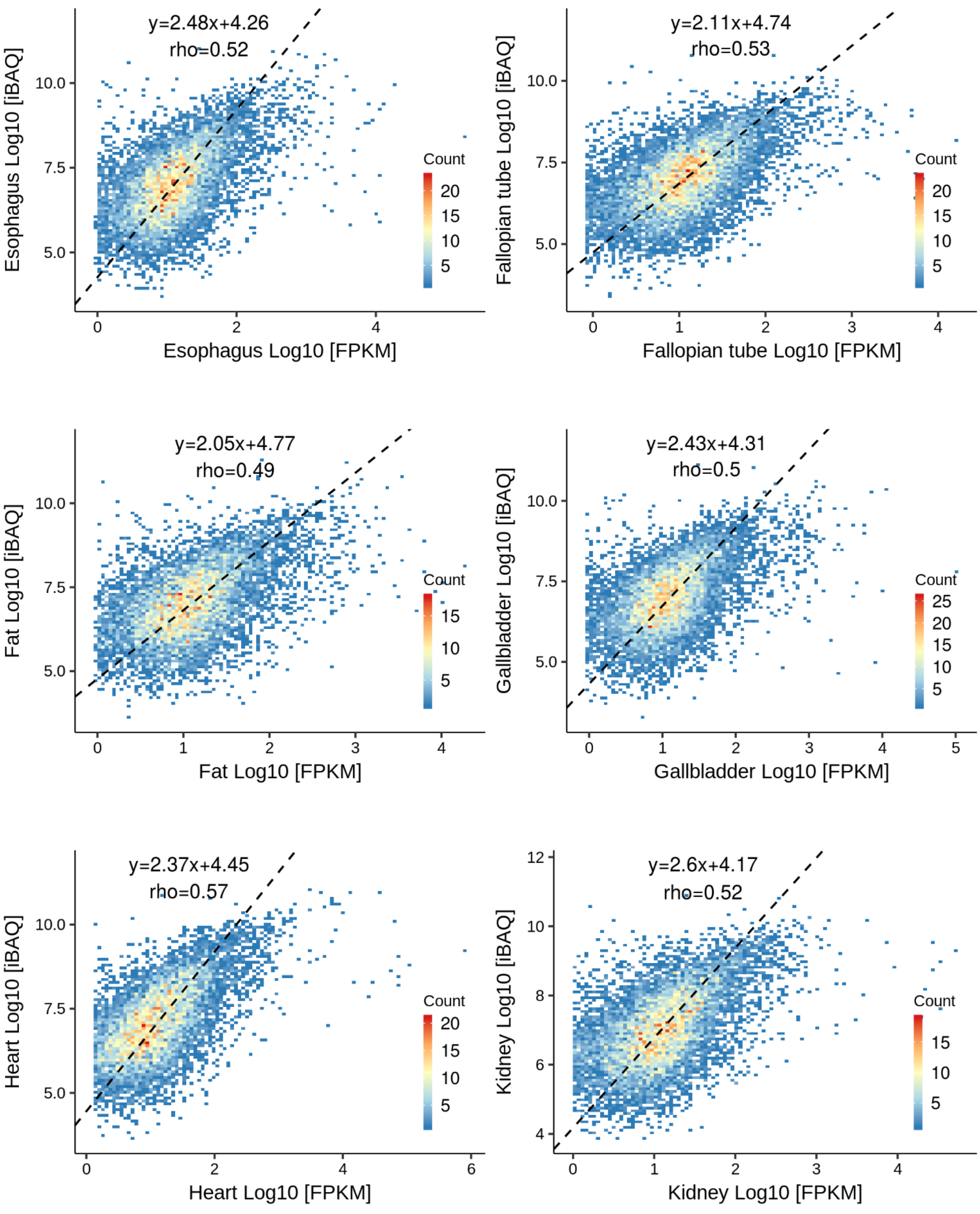
y=2.48x+4.26
rho=0.52
Esophagus Log10 [iBAQ]
Esophagus Log10 [FPKM]
Count
20
15
10
5
y=2.11x+4.74
rho=0.53
Fallopian tube Log10 [iBAQ]
Fallopian tube Log10 [FPKM]
Count
20
15
10
5
y=2.05x+4.77
rho=0.49
Fat Log10 [iBAQ]
Fat Log10 [FPKM]
Count
15
10
5
y=2.43x+4.31
rho=0.5
Gallbladder Log10 [iBAQ]
Gallbladder Log10 [FPKM]
Count
25
20
15
10
5
y=2.37x+4.45
rho=0.57
Heart Log10 [iBAQ]
Heart Log10 [FPKM]
Count
20
15
10
5
y=2.6x+4.17
rho=0.52
Kidney Log10 [iBAQ]
Kidney Log10 [FPKM]
Count
15
10
5

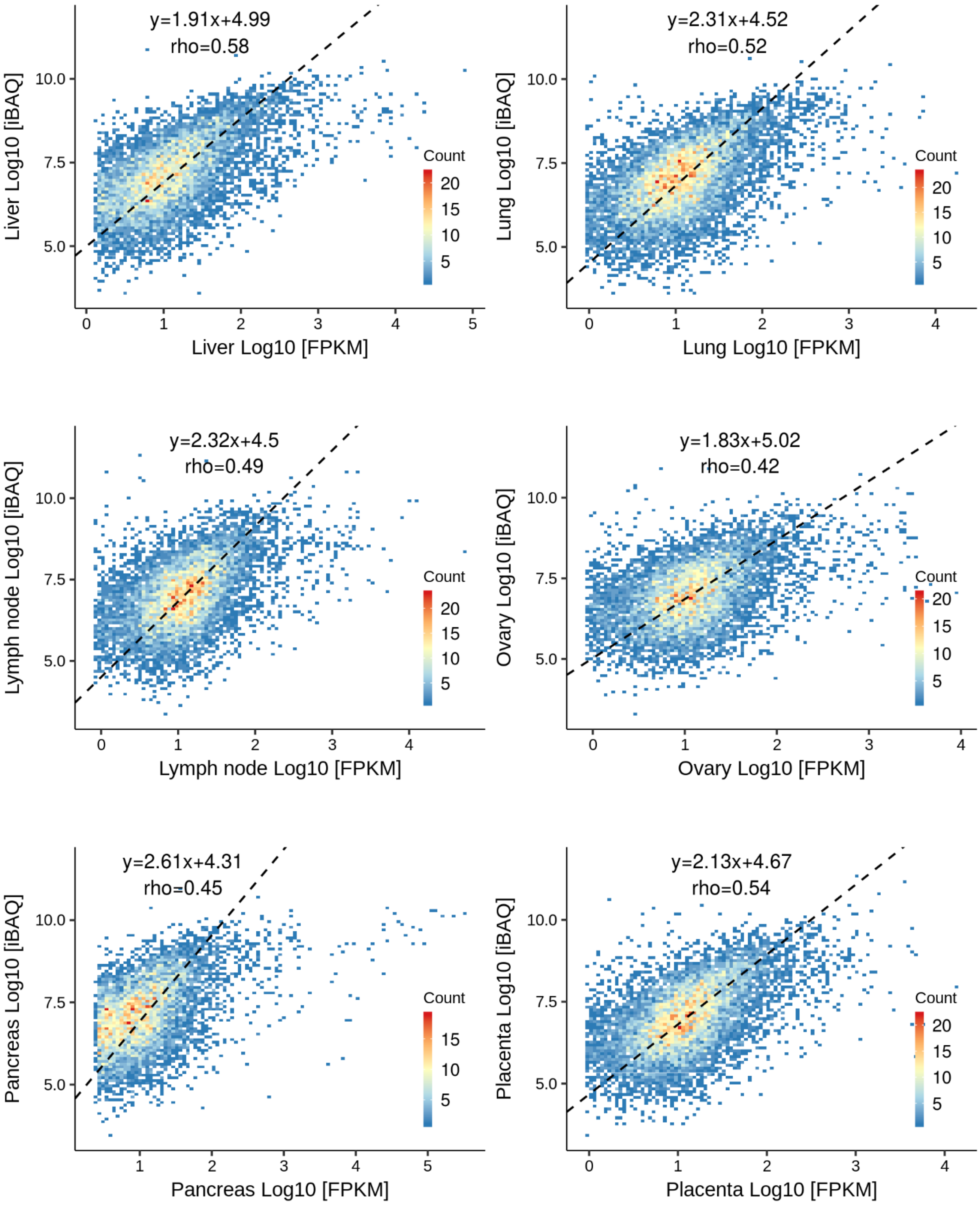
y=1.91x+4.99
rho=0.58
Liver Log10 [iBAQ]
Liver Log10 [FPKM]
Count
y=2.31x+4.52
rho=0.52
Lung Log10 [iBAQ]
Lung Log10 [FPKM]
y=2.32x+4.5
rho=0.49
Lymph node Log10 [iBAQ]
Lymph node Log10 [FPKM]
y=1.83x+5.02
rho=0.42
Ovary Log10 [iBAQ]
Ovary Log10 [FPKM]
y=2.61x+4.31
rho=0.45
Pancreas Log10 [iBAQ]
Pancreas Log10 [FPKM]
y=2.13x+4.67
rho=0.54
Placenta Log10 [iBAQ]
Placenta Log10 [FPKM]

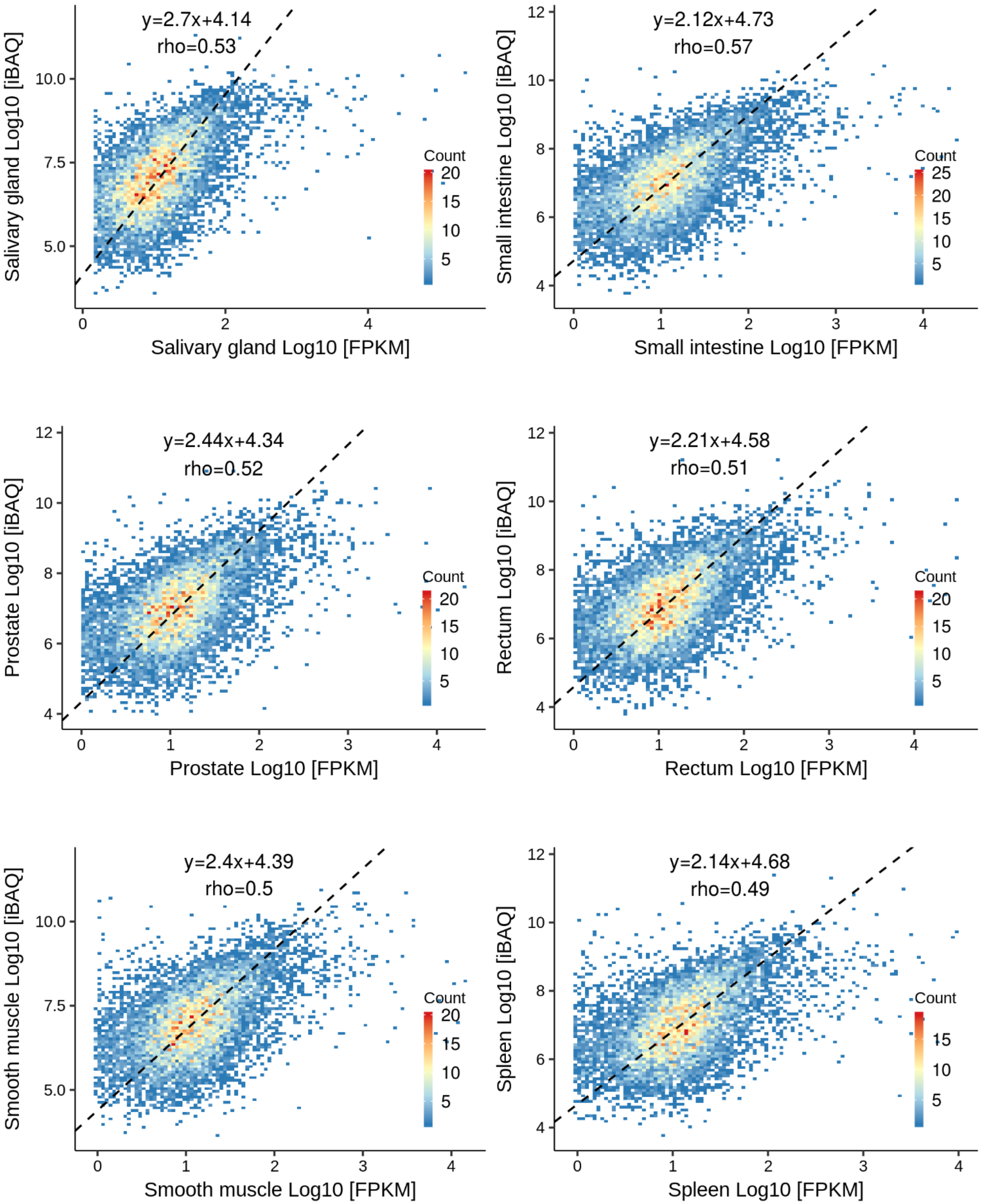
y=2.7x+4.14
rho=0.53
Salivary gland Log10 [iBAQ]
Salivary gland Log10 [FPKM]
Count
y=2.12x+4.73
rho=0.57
Small intestine Log10 [iBAQ]
Small intestine Log10 [FPKM]
y=2.44x+4.34
rho=0.52
Prostate Log10 [iBAQ]
Prostate Log10 [FPKM]
y=2.21x+4.58
rho=0.51
Rectum Log10 [iBAQ]
Rectum Log10 [FPKM]
y=2.4x+4.39
rho=0.5
Smooth muscle Log10 [iBAQ]
Smooth muscle Log10 [FPKM]
y=2.14x+4.68
rho=0.49
Spleen Log10 [iBAQ]
Spleen Log10 [FPKM]

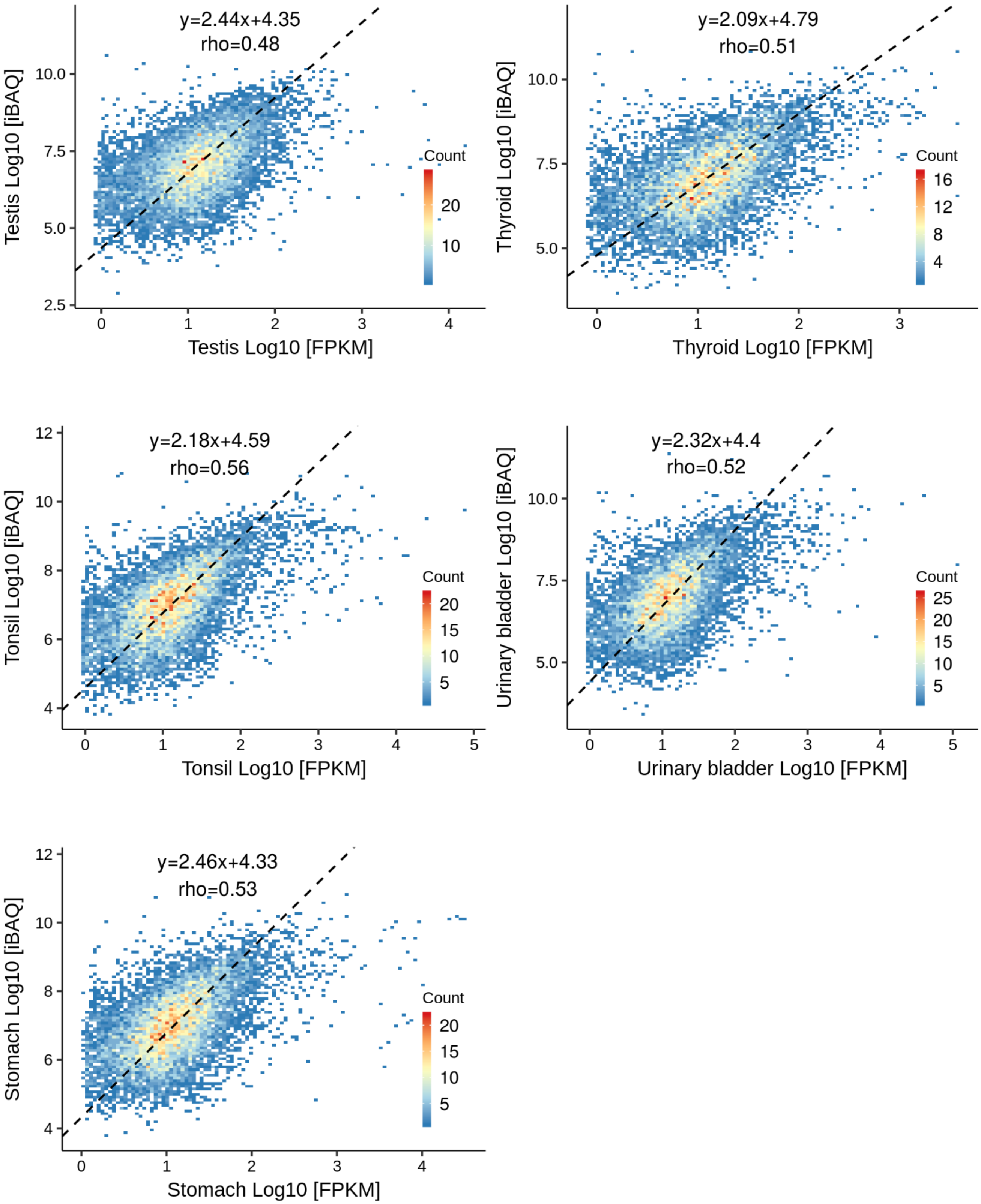
y=2.44x+4.35
rho=0.48
Testis Log10 [iBAQ]
Testis Log10 [FPKM]
Count
y=2.09x+4.79
rho=0.51
Thyroid Log10 [iBAQ]
Thyroid Log10 [FPKM]
Count
y=2.18x+4.59
rho=0.56
Tonsil Log10 [iBAQ]
Tonsil Log10 [FPKM]
Count
y=2.32x+4.4
rho=0.52
Urinary bladder Log10 [iBAQ]
Urinary bladder Log10 [FPKM]
Count
y=2.46x+4.33
rho=0.53
Stomach Log10 [iBAQ]
Stomach Log10 [FPKM]
Count

Appendix Figure S12. Rank abundance distribution of all proteins detected each of the 29 tissues. The five most abundant transcripts and proteins of each tissue are labled.

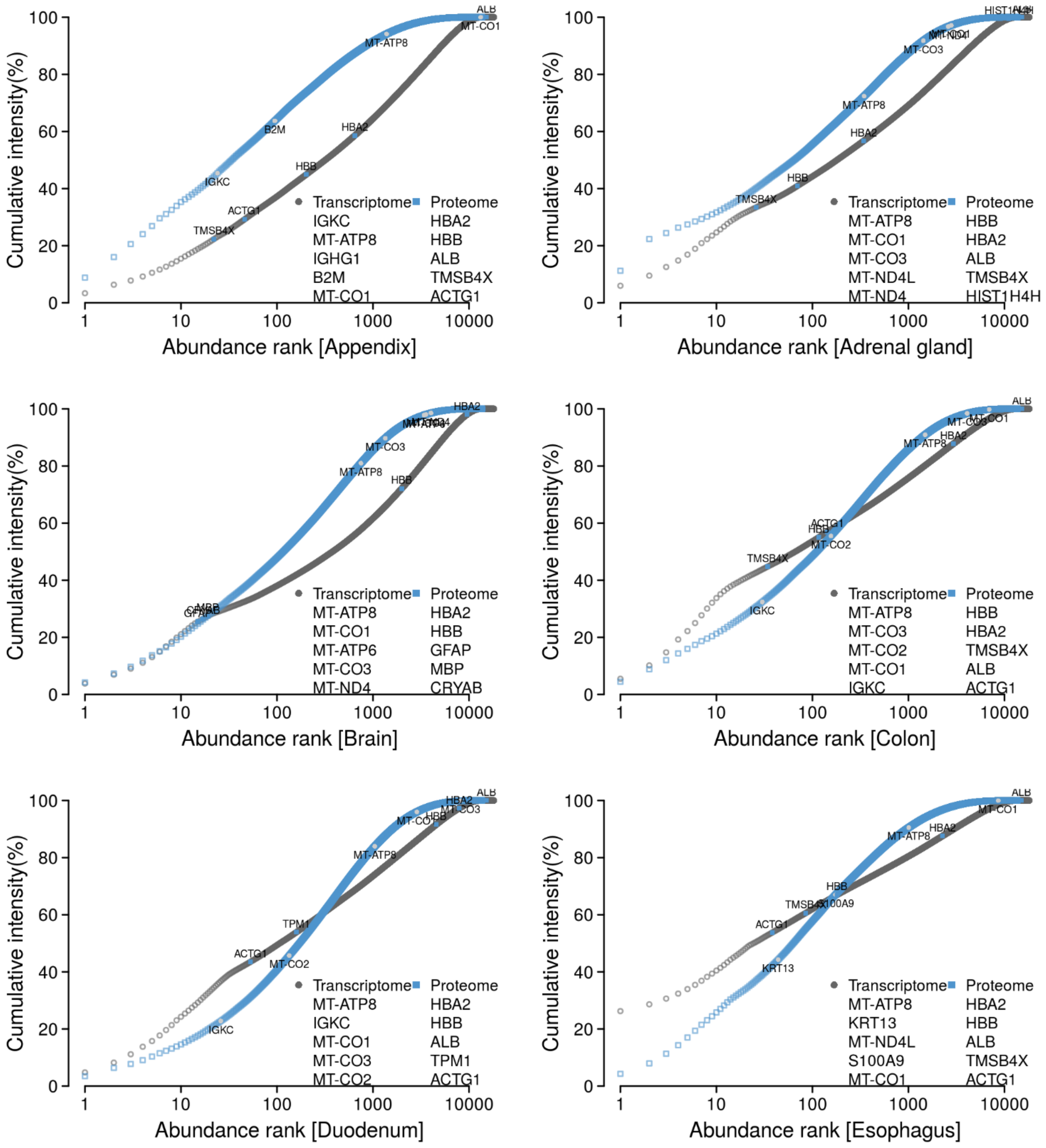

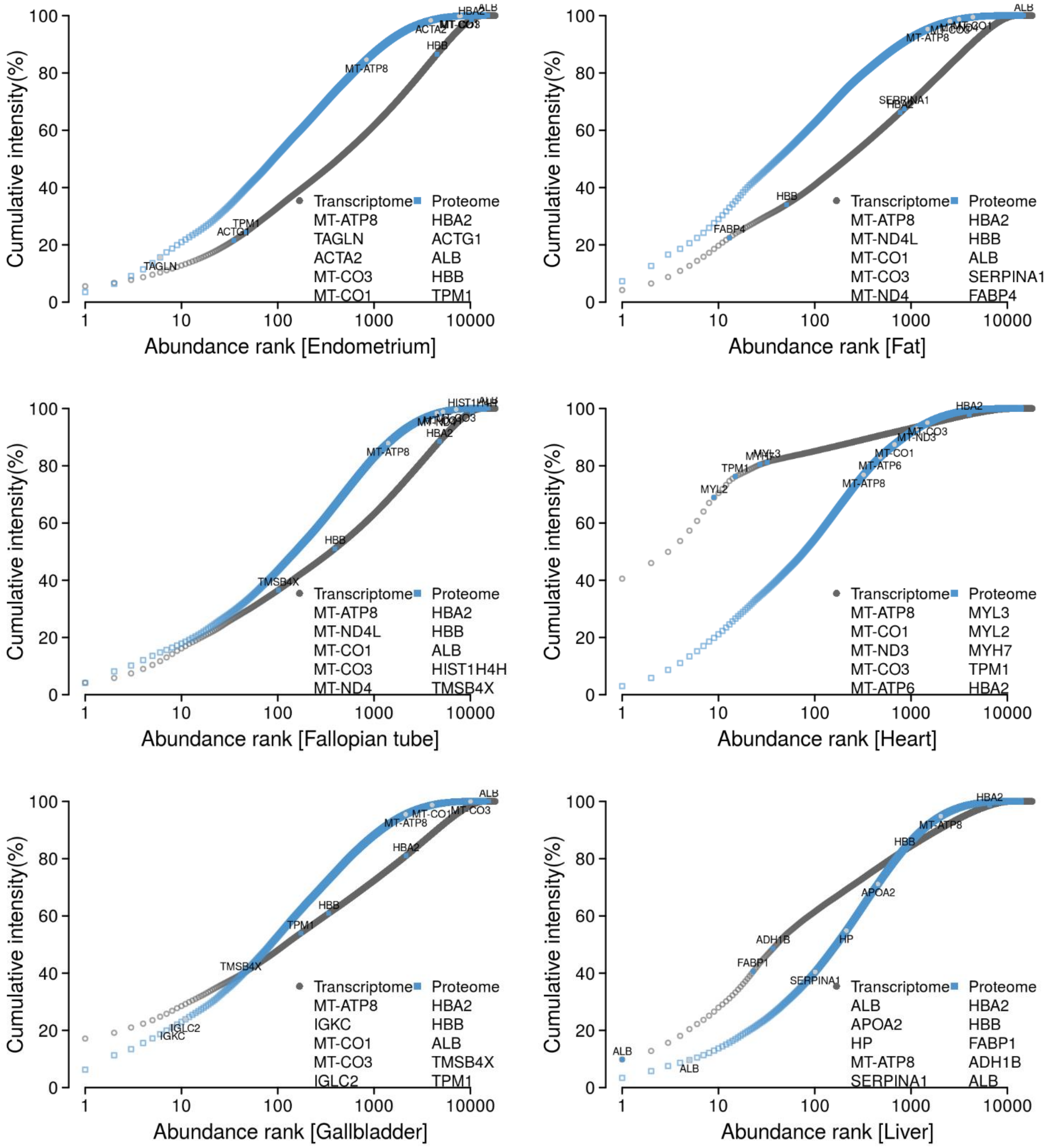
Cumulative intensity(%)
Abundance rank [Endometrium]
Transcriptome
MT-ATP8
TAGLN
ACTA2
MT-CO3
MT-CO1
Proteome
HBA2
ACTG1
ALB
HBB
TPM1
Abundance rank [Fat]
Transcriptome
MT-ATP8
MT-ND4L
MT-CO1
MT-CO3
MT-ND4
Proteome
HBA2
HBB
ALB
SERPINA1
FABP4
Abundance rank [Fallopian tube]
Transcriptome
MT-ATP8
MT-ND4L
MT-CO1
MT-CO3
MT-ND4
Proteome
HBA2
HBB
ALB
HIST1H4H
TMSB4X
Abundance rank [Heart]
Transcriptome
MT-ATP8
MT-CO1
MT-ND3
MT-CO3
MT-ATP6
Proteome
MYL3
MYL2
MYH7
TPM1
HBA2
Abundance rank [Gallbladder]
Transcriptome
MT-ATP8
IGKC
MT-CO1
MT-CO3
IGLC2
Proteome
HBA2
HBB
ALB
TMSB4X
TPM1
Abundance rank [Liver]
Transcriptome
ALB
APOA2
HP
MT-ATP8
SERPINA1
Proteome
HBA2
HBB
FABP1
ADH1B
ALB

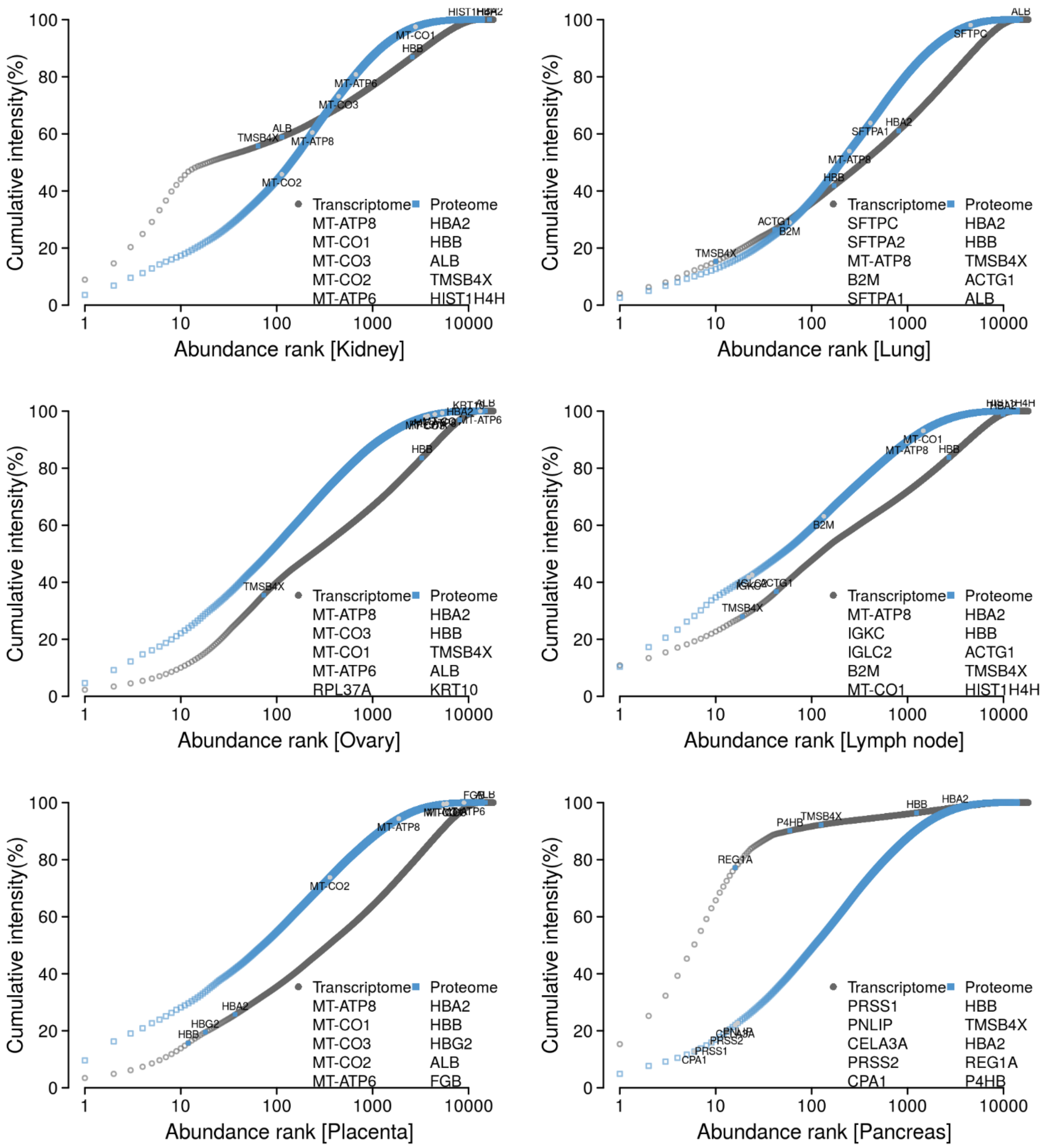
Cumulative intensity(%)
Abundance rank [Kidney]
Transcriptome
MT-ATP8
MT-CO1
MT-CO3
MT-CO2
MT-ATP6
Proteome
HBA2
HBB
ALB
TMSB4X
HIST1H4H
Abundance rank [Lung]
Transcriptome
SFTPC
SFTPA2
MT-ATP8
B2M
SFTPA1
Proteome
HBA2
HBB
TMSB4X
ACTG1
ALB
Abundance rank [Ovary]
Transcriptome
MT-ATP8
MT-CO3
MT-CO1
MT-ATP6
RPL37A
Proteome
HBA2
HBB
TMSB4X
ALB
KRT10
Abundance rank [Lymph node]
Transcriptome
MT-ATP8
IGKC
IGLC2
B2M
MT-CO1
Proteome
HBA2
HBB
ACTG1
TMSB4X
HIST1H4H
Abundance rank [Placenta]
Transcriptome
MT-ATP8
MT-CO1
MT-CO3
MT-CO2
MT-ATP6
Proteome
HBA2
HBB
HBG2
ALB
FGB
Abundance rank [Pancreas]
Transcriptome
PRSS1
PNLIP
CELA3A
PRSS2
CPA1
Proteome
HBB
TMSB4X
HBA2
REG1A
P4HB

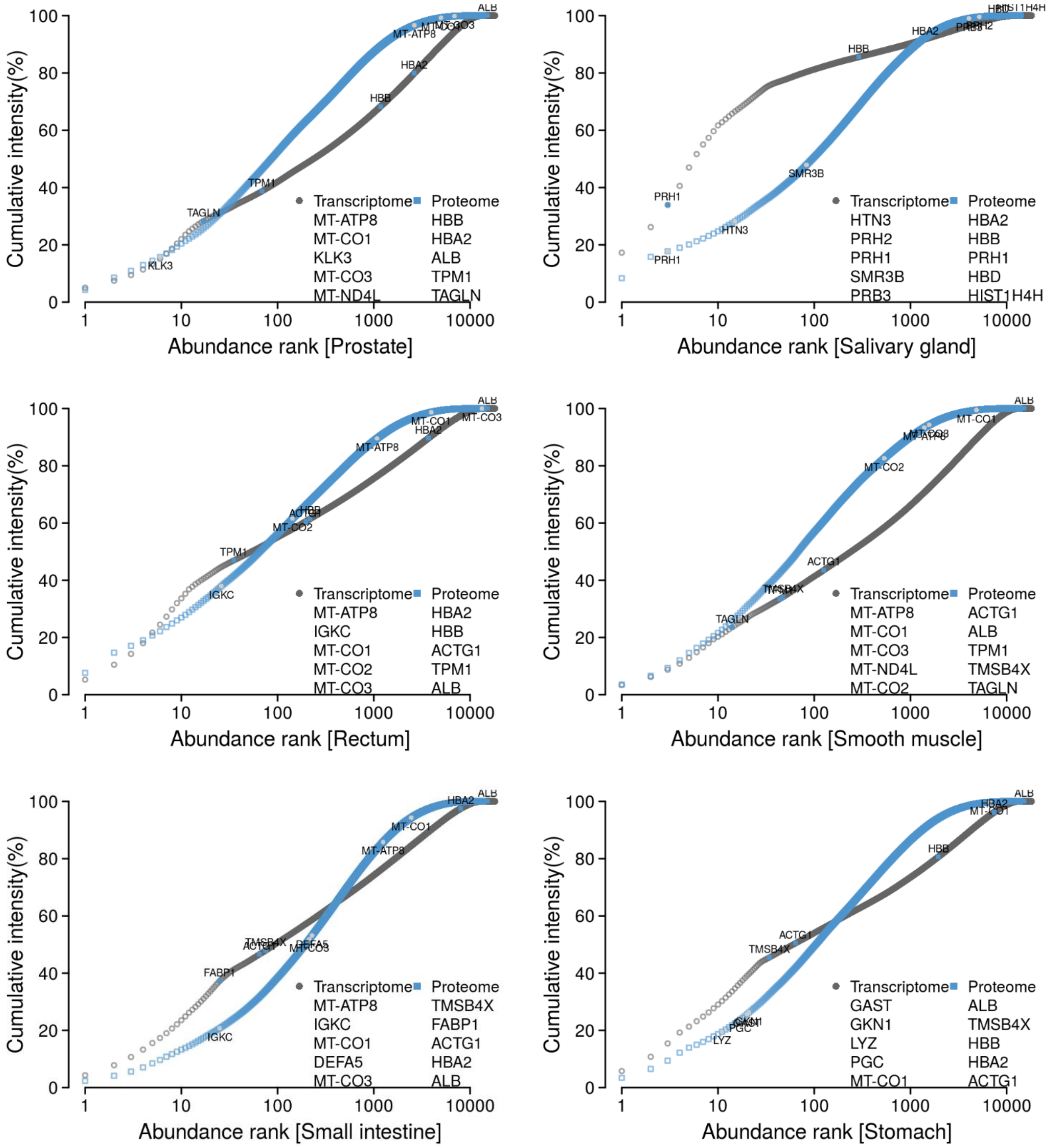
Cumulative intensity(%)
Abundance rank [Prostate]
Transcriptome
MT-ATP8
MT-CO1
KLK3
MT-CO3
MT-ND4L
Proteome
HBB
HBA2
ALB
TPM1
TAGLN
Abundance rank [Salivary gland]
Transcriptome
HTN3
PRH2
PRH1
SMR3B
PRB3
Proteome
HBA2
HBB
PRH1
HBD
HIST1H4H
Abundance rank [Rectum]
Transcriptome
MT-ATP8
IGKC
MT-CO1
MT-CO2
MT-CO3
Proteome
HBA2
HBB
ACTG1
TPM1
ALB
Abundance rank [Smooth muscle]
Transcriptome
MT-ATP8
MT-CO1
MT-CO3
MT-ND4L
MT-CO2
Proteome
ACTG1
ALB
TPM1
TMSB4X
TAGLN
Abundance rank [Small intestine]
Transcriptome
MT-ATP8
IGKC
MT-CO1
DEFA5
MT-CO3
Proteome
TMSB4X
FABP1
ACTG1
HBA2
ALB
Abundance rank [Stomach]
Transcriptome
GAST
GKN1
LYZ
PGC
MT-CO1
Proteome
ALB
TMSB4X
HBB
HBA2
ACTG1

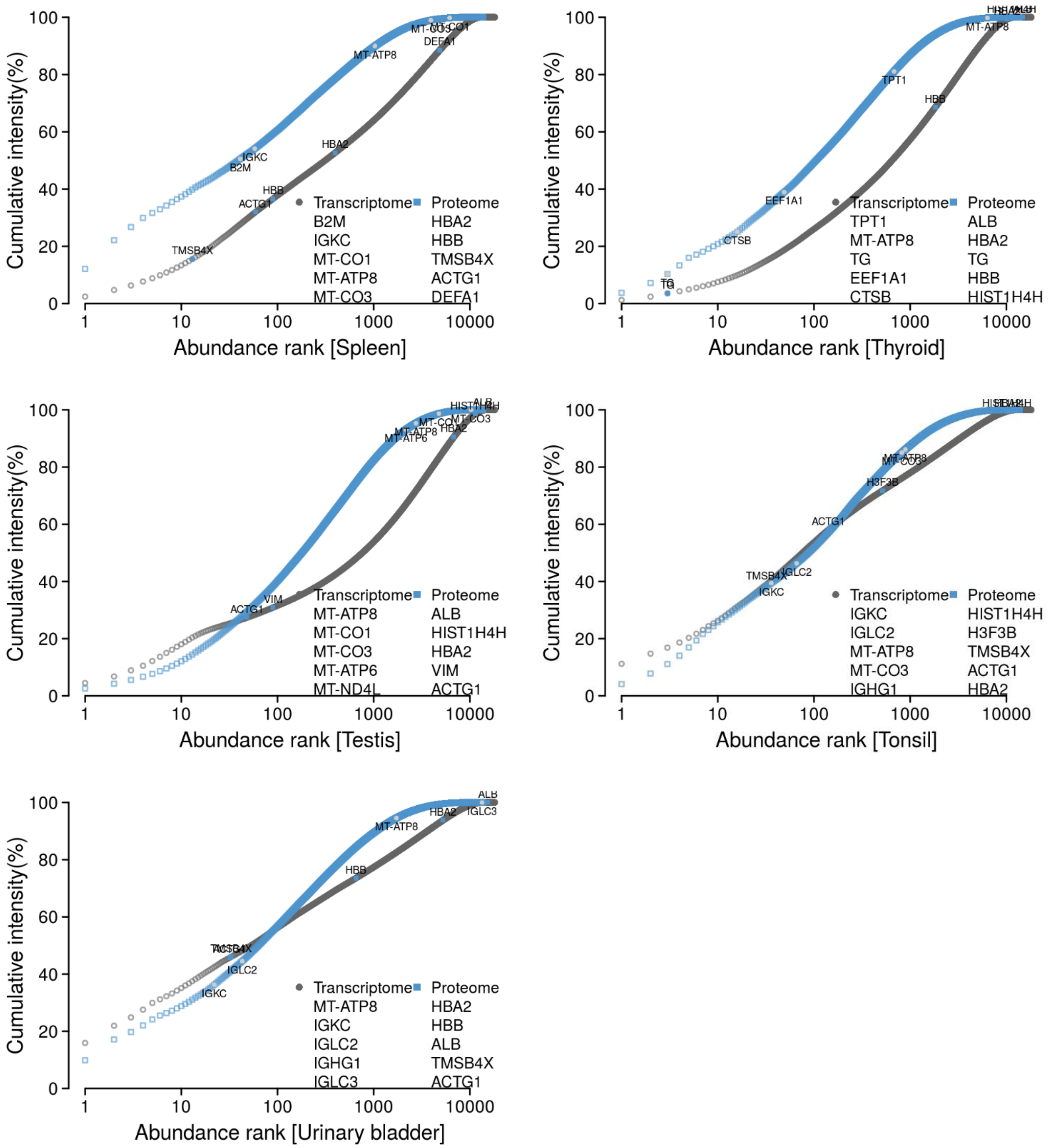
Cumulative intensity(%)
Abundance rank [Spleen]
Transcriptome
B2M
IGKC
MT-CO1
MT-ATP8
MT-CO3
Proteome
HBA2
HBB
TMSB4X
ACTG1
DEFA1
Abundance rank [Thyroid]
Transcriptome
TPT1
MT-ATP8
TG
EEF1A1
CTSB
Proteome
ALB
HBA2
TG
HBB
HIST1H4H
Abundance rank [Testis]
Transcriptome
MT-ATP8
MT-CO1
MT-CO3
MT-ATP6
MT-ND4L
Proteome
ALB
HIST1H4H
HBA2
VIM
ACTG1
Abundance rank [Tonsil]
Transcriptome
IGKC
IGLC2
MT-ATP8
MT-CO3
IGHG1
Proteome
HIST1H4H
H3F3B
TMSB4X
ACTG1
HBA2
Abundance rank [Urinary bladder]
Transcriptome
MT-ATP8
IGKC
IGLC2
IGHG1
IGLC3
Proteome
HBA2
HBB
ALB
TMSB4X
ACTG1

Appendix Figure S13. Distribution of protein vs transcript correlations ('gene-wise' correlation across 29 tissues). Only proteins expressed in at least 10 tissues were considered.

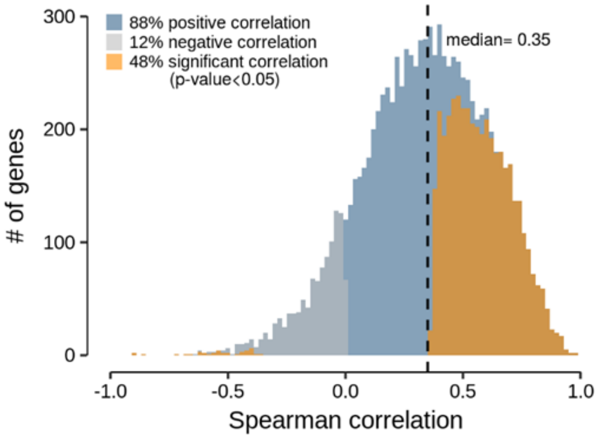

Appendix Figure S14. Distribution of protein vs transcript correlations ('gene-wise' correlation across 29 tissues). Only proteins expressed in at least 20 tissues were considered.

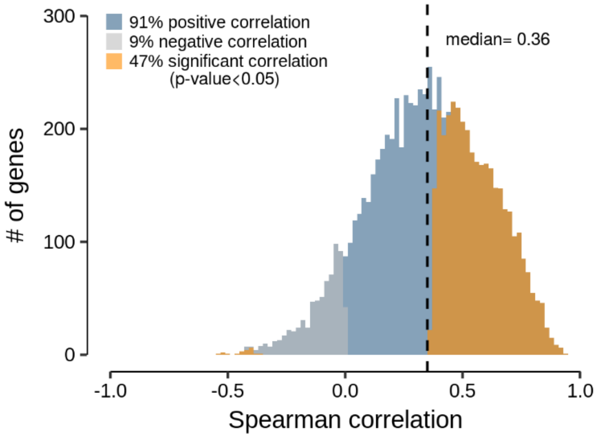

Appendix Figure S15. Distribution of protein vs transcript correlations ('gene-wise' correlation across 29 tissues) of all proteins. Only proteins expressed in all tissues were considered.

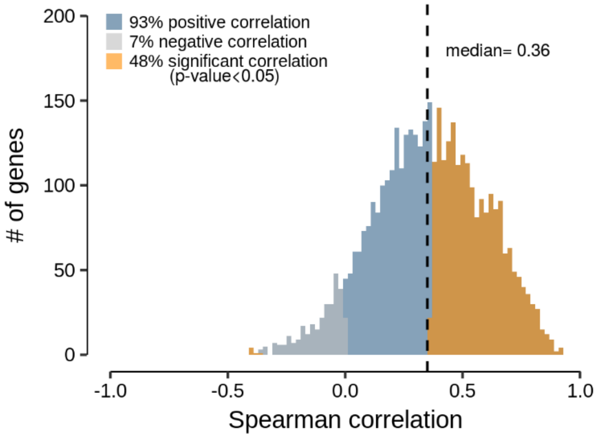

Appendix Figure S16. Comparison of gene-wise protein and transcript correlations and protein abundance. Only proteins expressed in at least 10 tissues were considered.

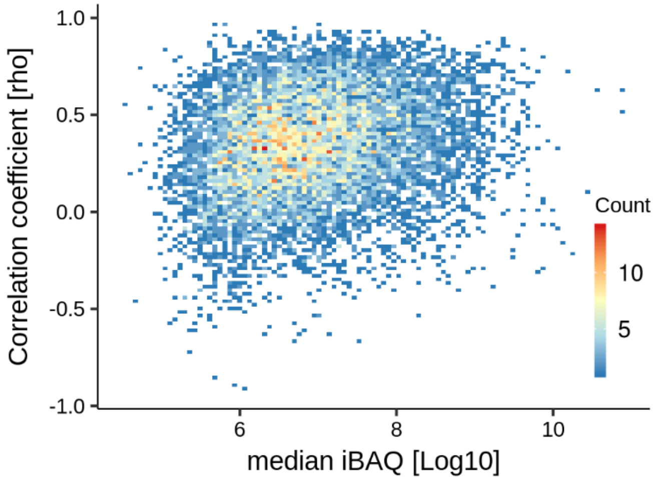

Appendix Figure S17. **Correlation analysis of transcripts and proteins of different levels of tissue-specificity classification.** Correlation analysis of all proteins and transcripts (A), proteins and transcripts with a mixed expression pattern (B), tissue-enhanced expression (C), group enriched expression (D), or tissue enriched expression (E). NA: grey

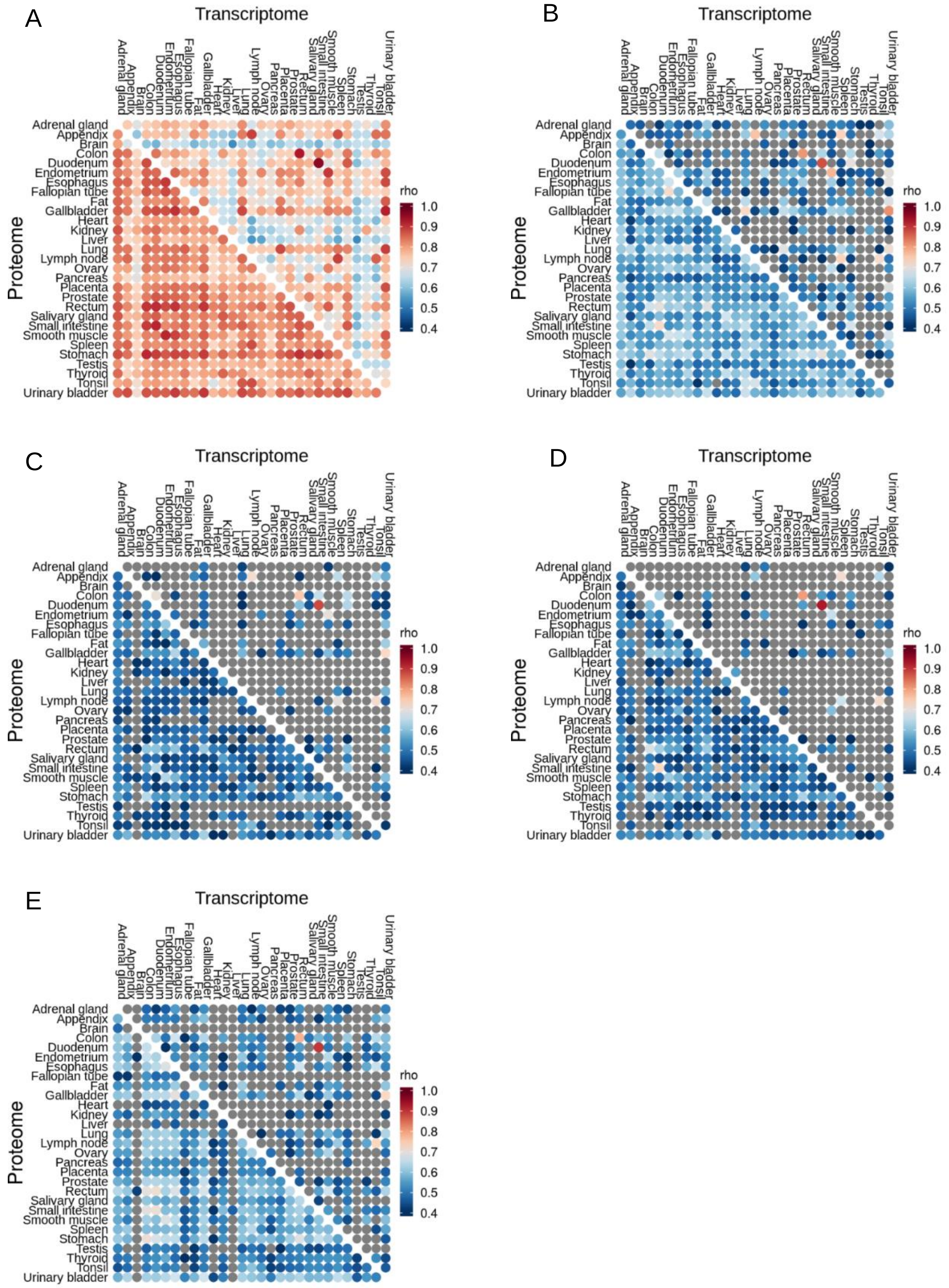

Appendix Figure S18. Influence of the sequence database on protein grouping. All 29 tissue proteomes were search against a human Ensembl protein sequence database (Ensembl, grey) as well as against a RNA-Seq derived tissue-specific database (RNA-Seq, blue).

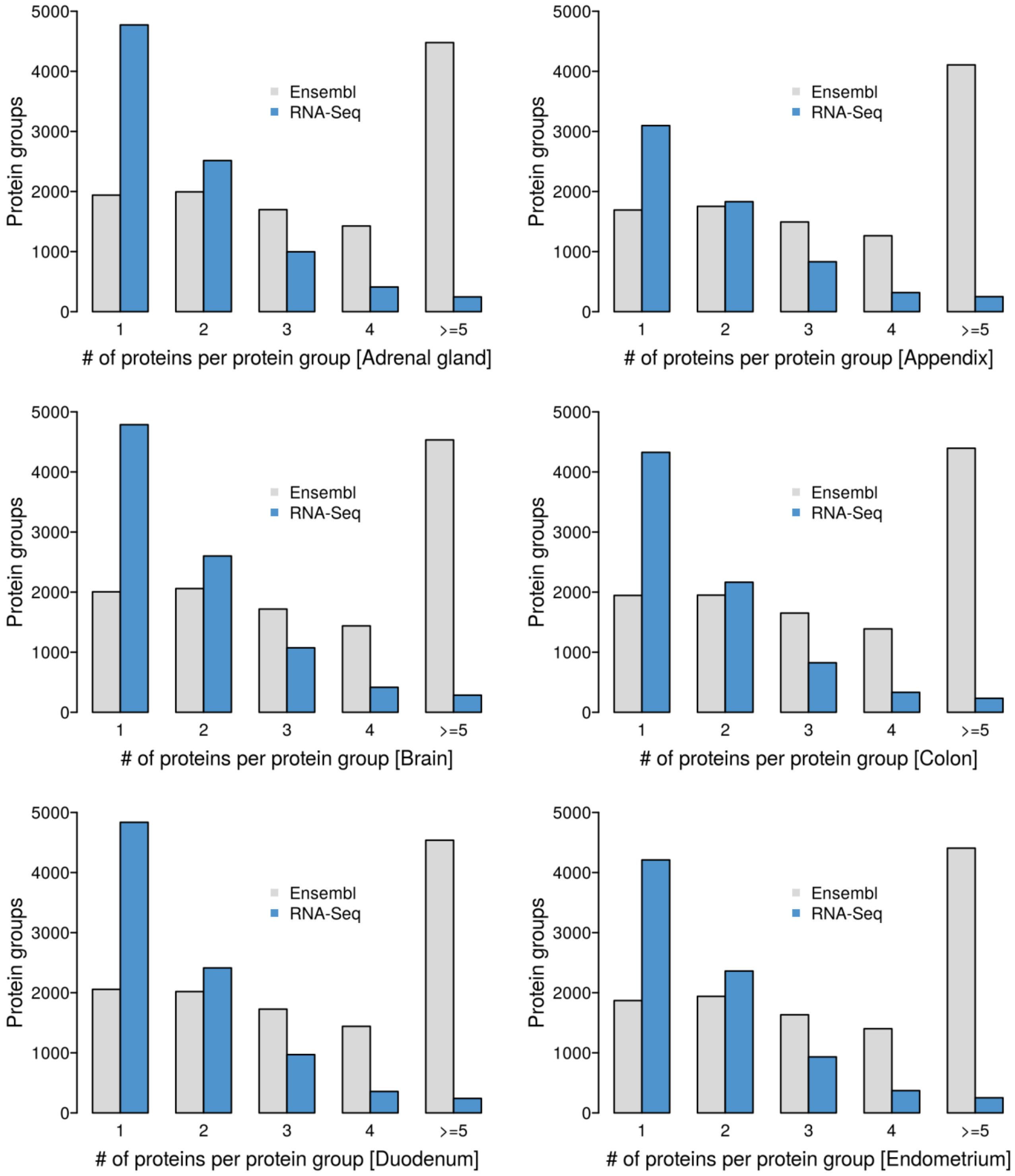

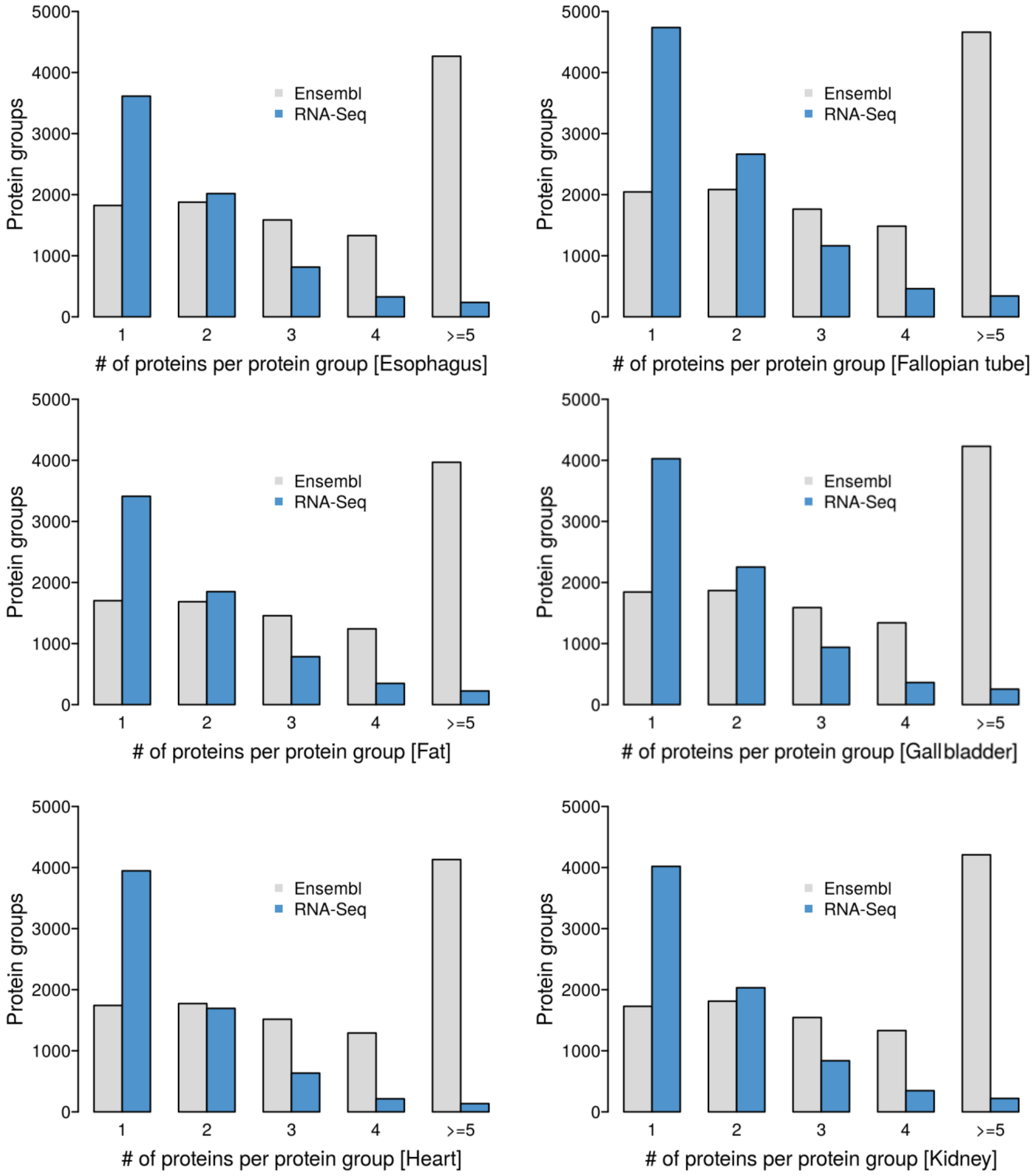
Protein groups
5000
4000
3000
2000
1000
0
Ensembl
RNA-Seq
1
2
3
4
>=5
# of proteins per protein group [Esophagus]
# of proteins per protein group [Fallopian tube]
# of proteins per protein group [Fat]
# of proteins per protein group [Gallbladder]
# of proteins per protein group [Heart]
# of proteins per protein group [Kidney]

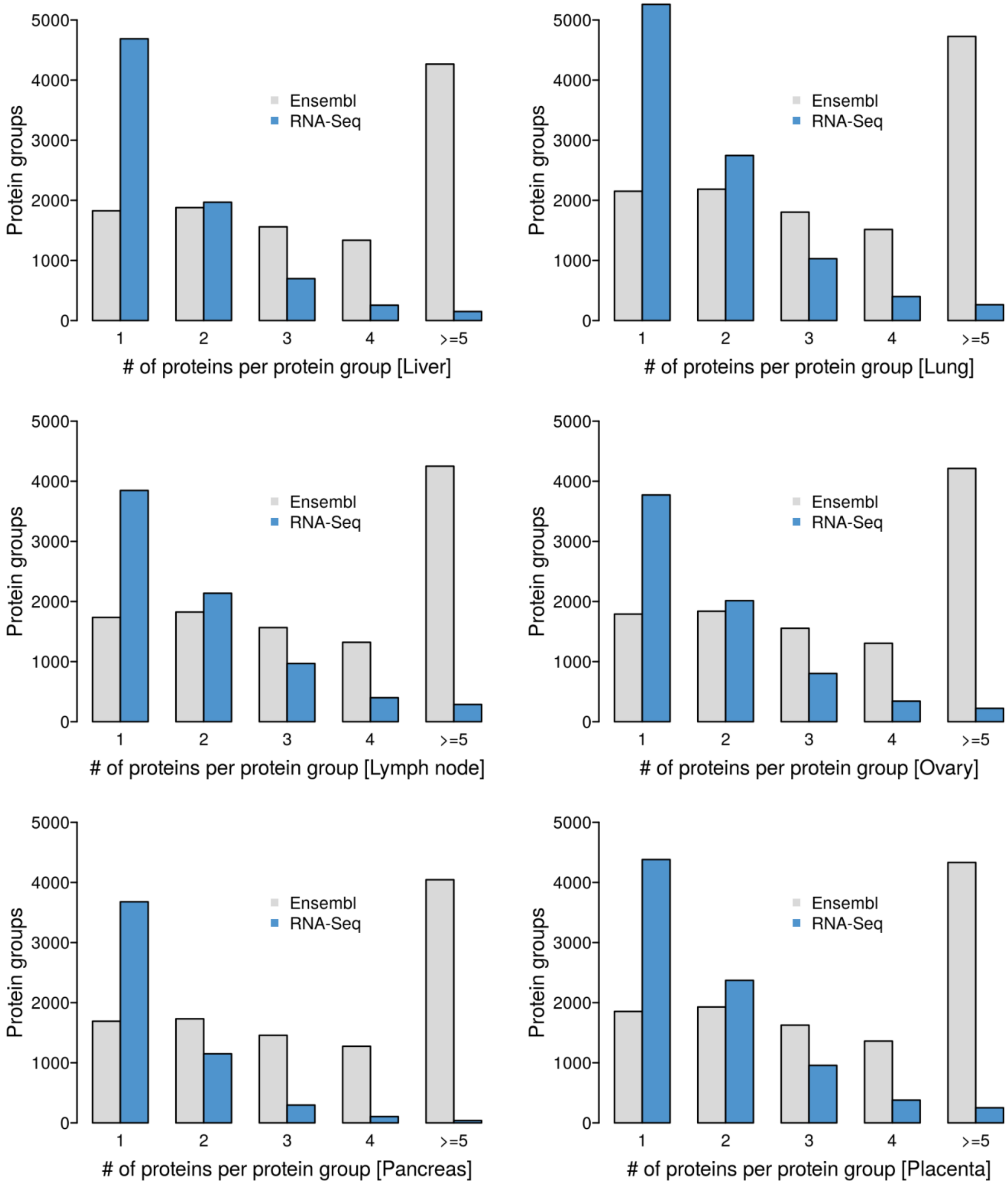
5000
4000
3000
2000
1000
0
Protein groups
Ensembl
RNA-Seq
1
2
3
4
>=5
# of proteins per protein group [Liver]
# of proteins per protein group [Lung]
# of proteins per protein group [Lymph node]
# of proteins per protein group [Ovary]
# of proteins per protein group [Pancreas]
# of proteins per protein group [Placenta]

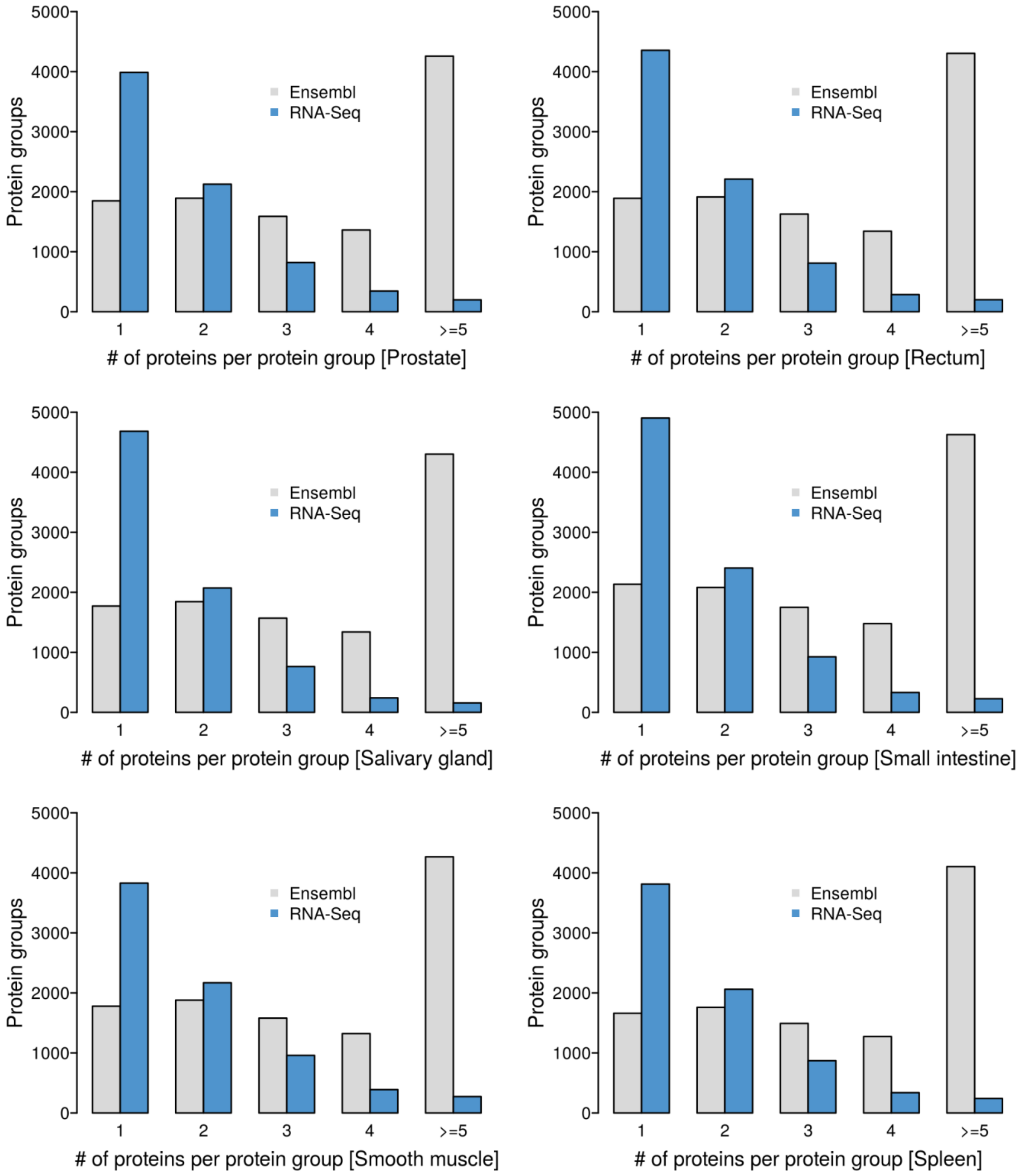
Protein groups
Ensembl
RNA-Seq
0
1000
2000
3000
4000
5000
1
2
3
4
>=5
# of proteins per protein group [Prostate]
# of proteins per protein group [Rectum]
# of proteins per protein group [Salivary gland]
# of proteins per protein group [Small intestine]
# of proteins per protein group [Smooth muscle]
# of proteins per protein group [Spleen]

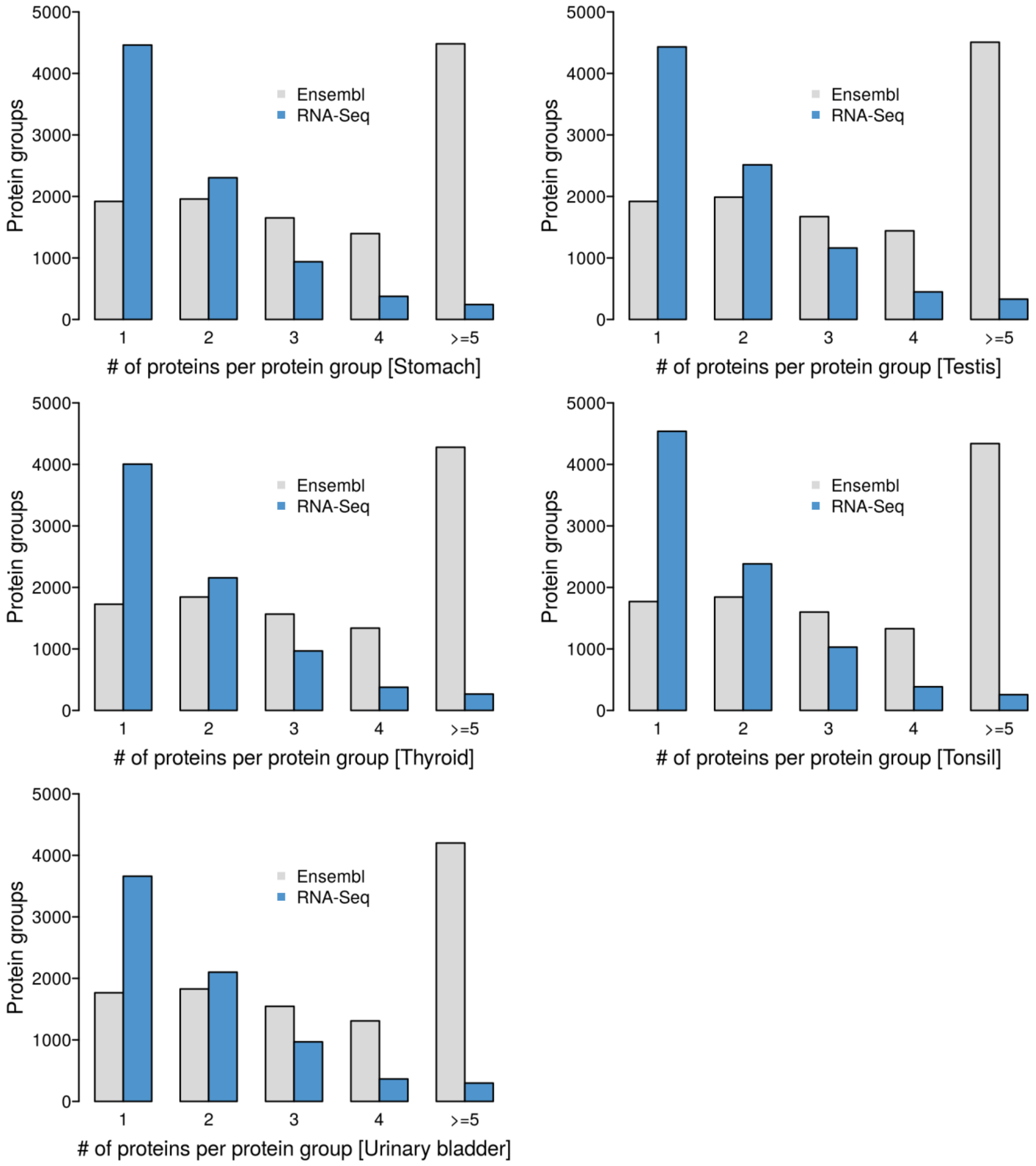
Protein groups
5000
4000
3000
2000
1000
0
Ensembl
RNA-Seq
1
2
3
4
>=5
# of proteins per protein group [Stomach]
# of proteins per protein group [Testis]
# of proteins per protein group [Thyroid]
# of proteins per protein group [Tonsil]
# of proteins per protein group [Urinary bladder]

Appendix Figure S19. Isoform detection in the ultra-deep tonsil proteome and the deep trypsin/HCD tonsil proteome. Ultra-deep tonsil proteome: dark blue, “All proteases_RNA-Seq”; deep trypsin/HCD tonsil proteome: light blue, “Trypsin_RNA-Seq”.

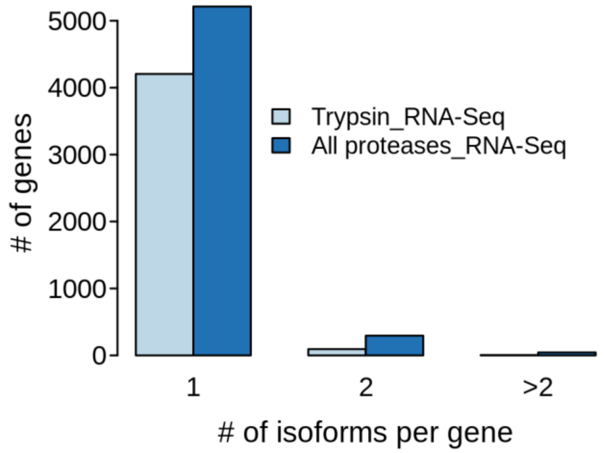

Appendix Figure S29. Experimental vs synthetic peptide reference spectra of candidate SAAV peptides identified by Mascot. The similarity of the experimental and reference spectrum was measured as spectrum contrast angle as well as Pearson correlation. Of each peptide, only the spectrum with highest spectral angle was plotted. Dotted lines mark similarity measures of 0.7, 0.8 and 0.9.

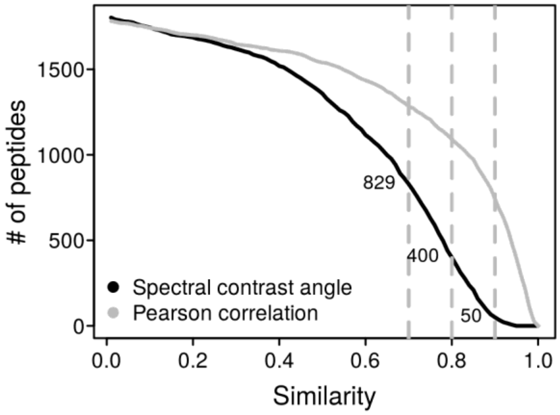

Appendix Figure S21. Experimental vs synthetic peptide reference spectra of candidate SAAV peptides identified by both Mascot and MaxQuant. The similarity of the experimental and reference spectrum was measured as spectrum contrast angle as well as Pearson correlation. Of each peptide, only the spectrum with highest spectral angle was plotted. Dotted lines mark similarity measures of 0.7, 0.8 and 0.9.

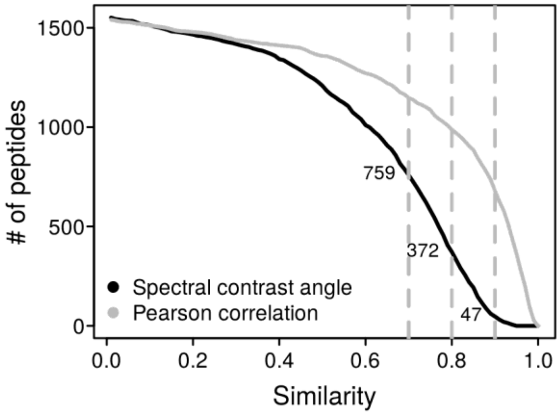

Supplement: Supplementary file 1 — Appendix [file MSB-15-e8503-s001.pdf]
