# Supplementary material for: A deep proteome and transcriptome abundance atlas of 29 healthy human tissues
Source: Mol Syst Biol. 2019 Feb 18;15(2):e8503. doi: 10.15252/msb.20188503 (PMC6379049; doi:10.15252/msb.20188503)
Supplement: Supplementary file 2 — Expanded View Figures PDF [file MSB-15-e8503-s002.pdf]

Expanded View Figures

Figure EV1. Further characterization of human proteomes and transcriptomes.

A Number of identified protein groups for each of the 29 tissues.
B Number of genes in all tissues that were detected at the transcript with higher than average expression but not detected at the protein level. Note the very high number of such cases in testis.
C Abundance distribution of all proteins detected in human brain (grey). Proteins in blue are expressed in all 29 tissues, and proteins in orange show elevated expression in brain.
D Clustering of gene ontology terms (biological process) for proteins and transcripts that show the most divergent expression across all tissue. Boxes give examples of GO terms for four different tissues (Appendix, brain, heart and testis).

© 2019 The Authors

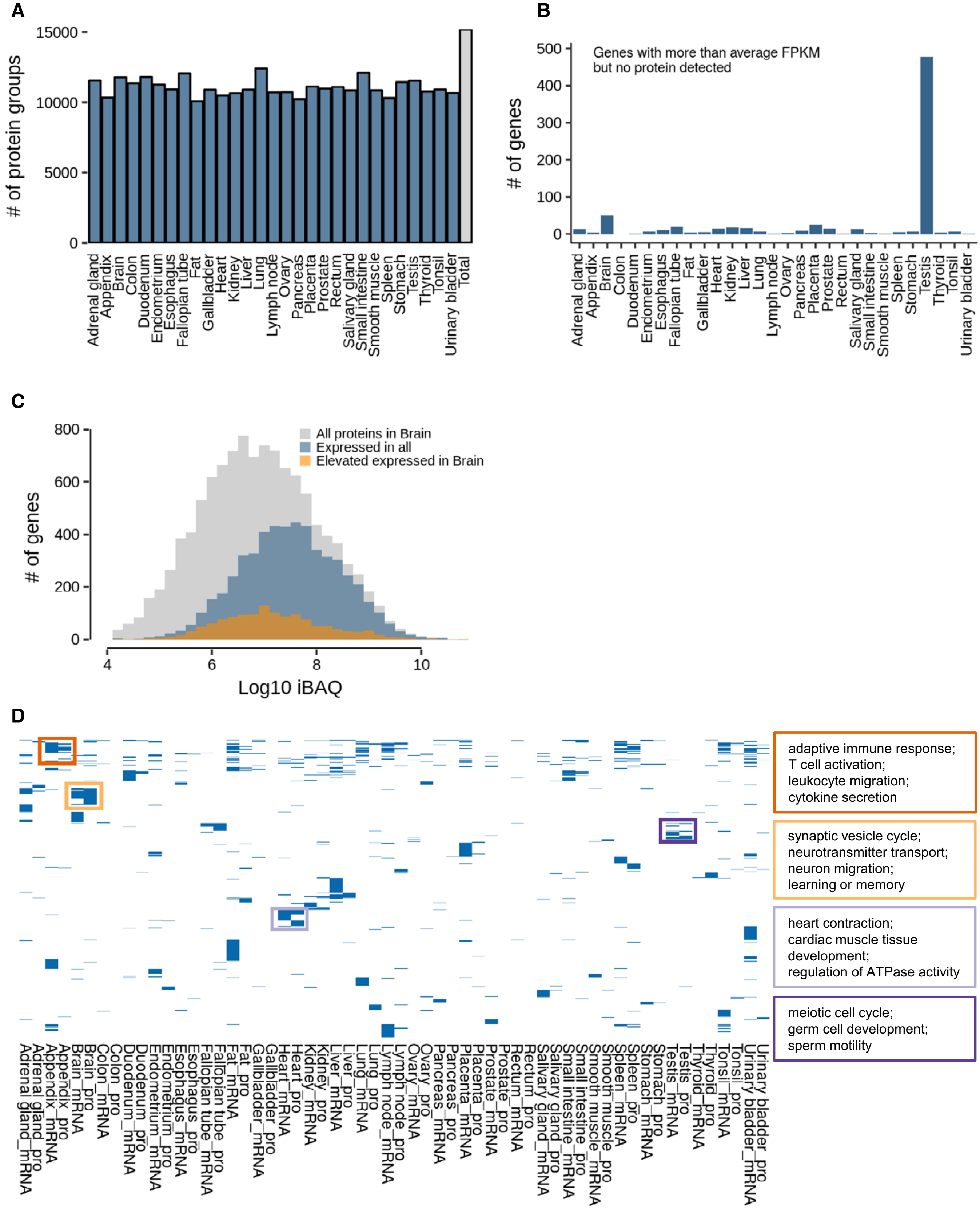

Figure EV1.

© 2019 The Authors

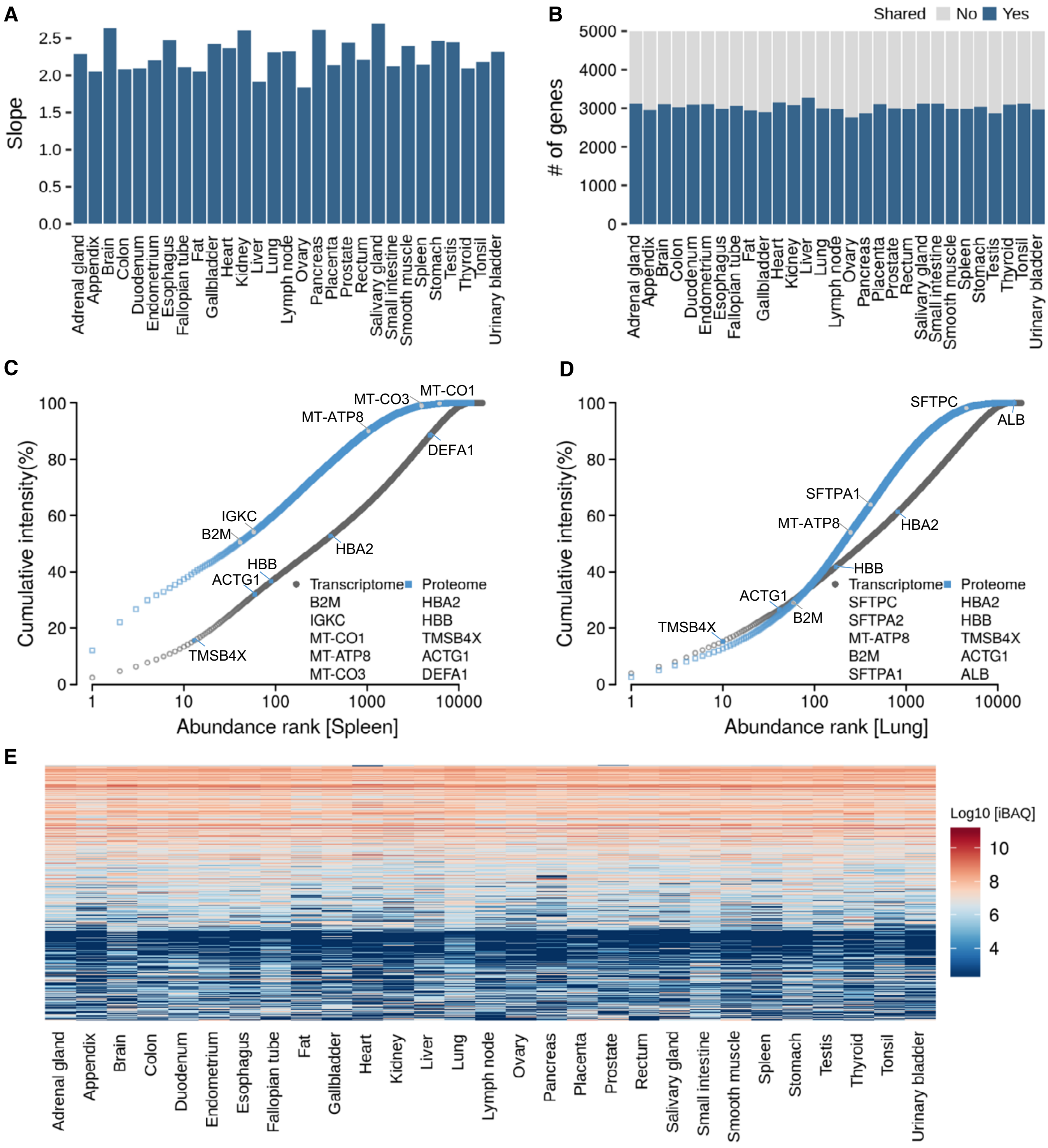

Figure EV2. Relationships between mRNA and protein expression.

A Slopes of the regression line in protein versus mRNA abundance plots (see main Fig 2B) for each tissue.

B Number of genes that are shared among the 5,000 most abundant transcripts or proteins in each tissue.

C, D Ranked abundance plots for transcripts and proteins in spleen and lung showing different characteristics in the abundance distributions (see also main Fig 2C and Appendix Fig S12 for all tissues).

E Clustering of protein abundances across all tissues. It is apparent that many proteins have similar expression levels across several/many tissues.

© 2019 The Authors

Figure EV3. Proteogenomic characterization of human tissues.

A Distribution of peptide sequence coverage values obtained for proteins by mass spectrometry in each tissue. Horizontal lines: median. Box limits: 50% quantiles. Whiskers: all data points outside the box.

B Analysis of the number of isoforms detected by transcriptomics or proteomics in each tissue.

C Distribution of peptide sequence coverage obtained for proteins by mass spectrometry in tonsil tissue broken down by protease and fragmentation method used. Horizontal lines: median. Box limits: 50% quantiles. Whiskers: all data points outside the box.

D Number of identified proteins in tonsil broken down by protease and fragmentation method used. Proteins covered by all workflows are marked in blue. Proteins identified by two or more workflows are indicated in grey and proteins exclusively identified by a single workflow in orange. The line connecting open circles indicates the cumulative number of proteins when adding data from the individual workflows.

E Same as panel (D) but for peptides. Peptides covered by more than one workflow are marked in grey, and those exclusive for one workflow are marked in orange.

F Number of experimental versus synthetic peptide reference spectra comparisons for candidate aTIS peptides (only the spectra with the highest spectral angle of each peptide were plotted) after database searching using Mascot as a function of the spectral angle or Pearson correlation coefficient. Dotted grey lines mark spectral angles of 0.7, 0.8 and 0.9.

G Same as panel (F) but showing only candidate peptides that were identified by both Mascot and Andromeda.

 © 2019 The Authors

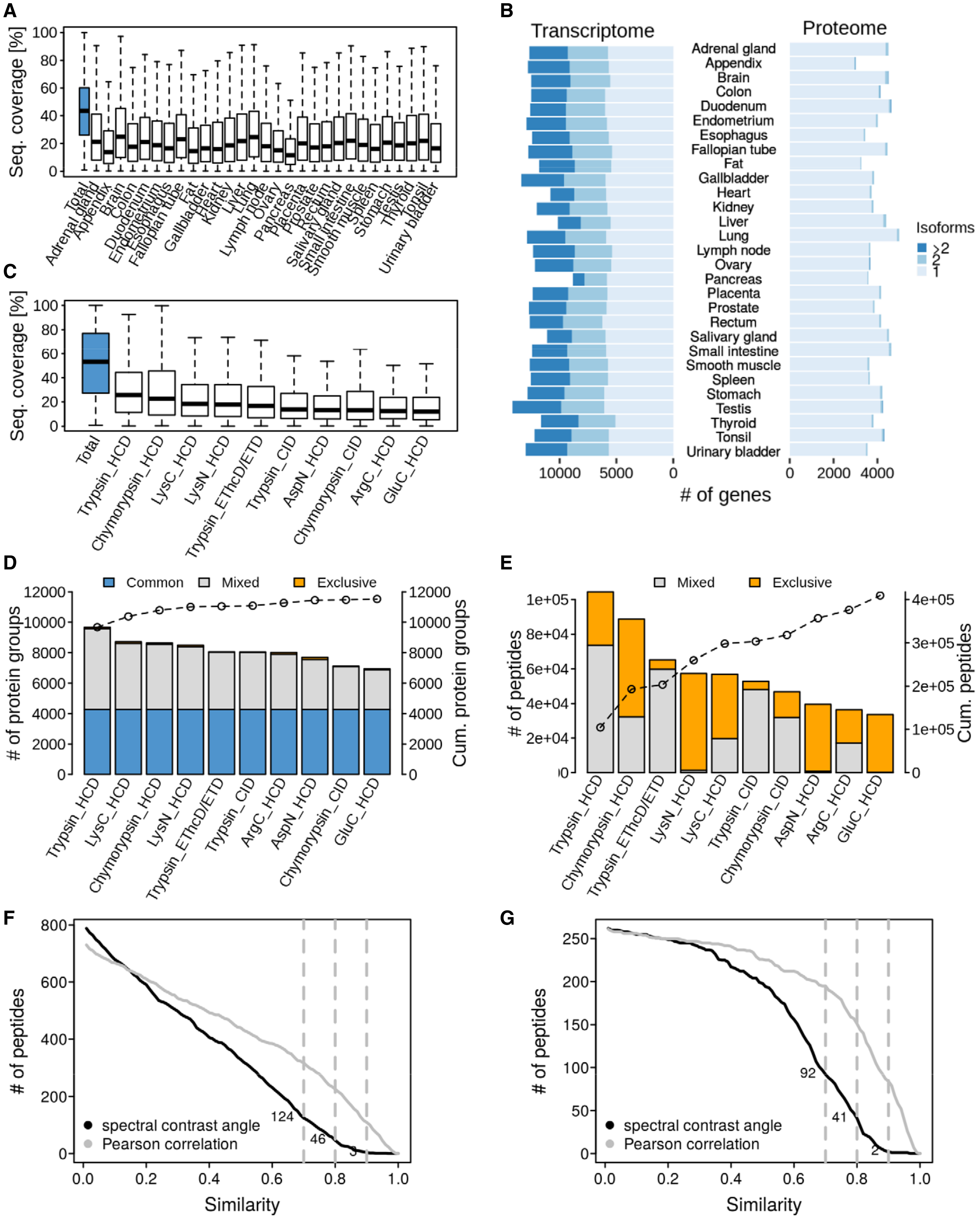
A
Seq. coverage [%]
Total
Adrenal gland
Appendix
Brain
Colon
Duodenum
Endometrium
Esophagus
Fallopian tube
Fat
Gallbladder
Heart
Kidney
Liver
Lung
Lymph node
Ovary
Pancreas
Placenta
Prostate
Rectum
Salivary gland
Small intestine
Smooth muscle
Spleen
Stomach
Testis
Thyroid
Tonsil
Urinary bladder
B
Transcriptome
Proteome
Isoforms
>2
2
1
# of genes
C
Seq. coverage [%]
Total
Trypsin_HCD
Chymorypsin_HCD
LysC_HCD
LysN_HCD
Trypsin_EThcD/ETD
Trypsin_CID
AspN_HCD
Chymorypsin_CID
ArgC_HCD
GluC_HCD
D
Common
Mixed
Exclusive
# of protein groups
Cum. protein groups
E
Mixed
Exclusive
# of peptides
Cum. peptides
F
# of peptides
spectral contrast angle
Pearson correlation
124
46
3
Similarity
G
# of peptides
92
41
2
spectral contrast angle
Pearson correlation
Similarity

Figure EV3.
